# Porphyrin-Based Bio-Sourced Materials for Water Depollution Under Light Exposure

**DOI:** 10.3390/polym17212882

**Published:** 2025-10-29

**Authors:** Fanny Schnetz, Marc Presset, Jean-Pierre Malval, Yamin Leprince-Wang, Isabelle Navizet, Davy-Louis Versace

**Affiliations:** 1 University Paris-Est Creteil, CNRS, ICMPE, UMR 7182, 94320 Thiais, France; fanny.schnetz@gmail.com (F.S.); marc.presset@cnrs.fr (M.P.); 2 Institut de Science des Matériaux de Mulhouse, UMR CNRS 7361, Université de Haute Alsace, 15 rue Jean Starcky, 68057 Mulhouse, France; jean-pierre.malval@uha.fr; 3 LGE, Université Gustave Eiffel, F-77454 Marne-la-Vallée, France; yamin.leprince@univ-eiffel.fr; 4 Univ Gustave Eiffel, Univ Paris Est Creteil, CNRS, UMR 8208, MSME, F-77454 Marne-la-Vallée, France; isabelle.navizet@univ-eiffel.fr

**Keywords:** photochemistry, photopolymerization, bio-based materials, EPR spin-trapping, reactive oxygen species, water depollution

## Abstract

The photoinitiation properties of two porphyrins were evaluated for the free-radical photopolymerization (FRP) of a bio-based acrylated monomer, i.e., soybean oil acrylate (SOA). Their combination with various co-initiators, such as a tertiary amine as electron donor (MDEA), an iodonium salt as electron acceptor (Iod), as well as two biosourced co-initiators used as H-donors (cysteamine and N-acetylcysteine), makes them highly efficient photoinitiating systems for FRP under visible light irradiation. Electron paramagnetic resonance spin trapping (EPR ST) demonstrated the formation of highly reactive radical species, and fluorescence and laser flash photolysis highlighted the chemical pathways followed by the porphyrin-based systems under light irradiation. High acrylate conversions up to 96% were obtained with these different systems at different irradiation wavelengths (LEDs@385 nm, 405 nm, 455 nm, and 530 nm), in laminate or under air. The final crosslinked and bio-based porphyrin-based materials were used for the full photo-oxidation in water of an azo-dye (acid red 14) and under UV irradiation. These materials have been involved in three successive depollution cycles without any reduction in their efficiency.

## 1. Introduction

Photopolymerization has received increasing attention in recent years [1] and is applied in a wide range of applications, including bioengineering [2], electronic components, and coatings [1]. The use of visible light to initiate polymerization has gained considerable attention, from both economic and environmental aspects [3,4,5]. The use of dyes to initiate polymerization, including eosin [6], natural dyes [7,8,9,10], anthracene [11], or phthalocyanines [12], makes it possible to produce photopolymerized materials under low-energy irradiation. However, despite their high absorption of visible light, porphyrins are not commonly used. In addition to their ability to initiate polymerization of various monomers by free radical or cationic polymerization [13,14,15,16,17,18,19] as well as PET-RAFT polymerization [20,21,22,23], porphyrins are known to react with oxygen present in the environment to generate reactive oxygen species [24,25,26,27], which are highly oxidative species. Coatings using porphyrins have already been used by our group to produce antibacterial materials [15,18], but the photoinduced properties of these materials could also have some applications concerning water depollution [28,29].

Synthetic dyes are widely employed in industries such as textiles, printing, dyeing, and food processing, and they cover various families, including azo, nitro, and indigo compounds. The textile industries, in particular, have experienced a marked rise in the use of complex synthetic organic dyes, which act as coloring agents. However, these dyes are only partially absorbed by fibers, resulting in highly colored wastewater that poses serious environmental challenges [30]. Industrial effluents often contain stable organic pollutants that are toxic to aquatic organisms and potentially carcinogenic to humans [31]. In addition, chemical transformations such as oxidation or hydrolysis may generate harmful by-products, further threatening ecosystems [32]. In some cases, dyes or their degradation products can even infiltrate drinking water supplies, raising public health concerns [31]. Eliminating these dyes before wastewater discharge is therefore crucial, but their degradation—especially that of azo dyes, which account for roughly 70% of global usage—is difficult due to their synthetic origin and structural complexity [33]. Azo dyes are characterized by an azo bond (–N=N–), which, together with other chromophores, is responsible for their coloration [34,35]. Traditional treatment strategies, such as membrane separation [36], adsorption [37], extraction [38], and electrodialysis processes [39], have been applied to remove these contaminants. However, most of these methods are not destructive; they simply transfer pollutants to another phase, creating secondary waste streams that demand further treatment [40,41,42]. Moreover, these approaches are often costly, inefficient, or produce additional toxic residues.

As an alternative, advanced oxidation processes (AOPs) have gained increasing attention. These methods rely on the in situ generation of highly reactive species such as hydroxyl radicals (•OH), which can mineralize a wide range of organic compounds in wastewater [43,44]. AOPs are considered promising for textile effluents because of their high efficiency, cost-effectiveness, and potential recyclability [45,46]. Among them, Fenton chemistry—based on hydrogen peroxide and ferrous ions—has been widely studied [47]. While effective, homogeneous Fenton processes are constrained by the need for acidic conditions (pH 2–4) and the large quantities of iron-rich sludge generated, which increase operational costs and environmental burden [48]. Heterogeneous Fenton systems, such as iron-loaded clays or zeolites [49,50,51,52], have been proposed to overcome these drawbacks, showing improved efficiency and easier handling. Recent research has also explored elemental doping and hybrid catalysts ([(Ni, Mg, Cu)Fe_2_O_4_] [53], hybrid materials (e.g., α-FeOOH@GCA [54], Cd/GO/Fe_2_O [55] or Fe_2_O_3_@GO [56] to enhance photo-Fenton activity. Despite these advances, issues such as iron leaching and activity limited to acidic pH remain barriers to practical use. In the same way, materials based on metal oxide semiconductors like TiO_2_ [57,58,59,60,61,62,63,64] have been used and demonstrated their efficiency in the degradation of azo pollutants; however, due to the uncertainty regarding the safety of nanoparticles, new solutions have been explored. To address these challenges, porous organic polymers (POPs) are emerging as a promising class of materials for catalytic applications [65,66]. POPs combine high surface area, tunable active sites, interconnected porosity that facilitates mass transfer, and favorable light absorption properties [65,66]. Incorporating metalloporphyrins as building units further enhances stability and catalytic efficiency thanks to their robust structures and versatile nitrogen functionalities [67,68]. Nonetheless, achieving uniform metal loading and strong interactions with the polymer matrix remains a challenge, highlighting the need for further innovation in catalyst design. However, the amount of porphyrin used for the synthesis of such materials has raised a crucial cost issue.

To enhance the biobased content of the photocatalytic materials and reduce their toxicity, biobased polymers incorporating porphyrins could be a promising alternative for effective degradation of dyes. Surprisingly, free-metal-based porphyrins have been scarcely used, although they are stable under light exposure irradiation [62,63]. Thus, the use of bio-based polymers incorporating smaller amounts of porphyrin could significantly reduce synthesis costs and environmental impact, particularly compared with studies involving metal oxides or porphyrin-based polymers. The originality of this study is in designing two new porphyrins, which will be used as photoinitiators for the free-radical polymerization (FRP) of a bio-based monomer, soybean oil acrylate (SOA), and as photosensitizers for the photo-oxidation of a water pollutant, i.e., acid red 14 (AR14). Under light activation, the porphyrin-based polymer materials could generate reactive oxygen species (ROS) that may oxidize AR14 in water. The properties of both porphyrins will be computed by density functional theory (DFT) and time-dependent density functional theory (TD-DFT) to determine their ground state structure and their theoretical absorbance properties. The photochemical properties of both porphyrins when combined with various co-initiators, i.e., electron donor (tertiary amine), acceptor (iodonium salt), or H-donor molecules (i.e., cysteamine or N-acetylcysteine), will be studied in detail by steady state photolysis, fluorescence, laser flash photolysis, and electronic paramagnetic resonance spin-trapping (EPR-ST). The photoinitiating properties of the porphyrin-like systems will be evaluated through the FRP of SOA under low-intensity blue LEDs (405 and 455 nm) and green LED (530 nm) irradiation and followed by real-time Fourier-transformed infrared spectroscopy (RT-FTIR). The final photoinduced and crosslinked materials will be used to perform the photo-oxidation of AR14 in water under light exposure, and their reusability will be demonstrated.

## 2. Materials and Methods

Materials. Solvents (acetic acid, methanol, dichloromethane = DCM, petroleum ether, and acetone) were used as received. Tetrahydrofuran (THF) for Suzuki–Miyaura coupling reactions was distilled under argon using sodium and benzophenone. Pyrrole (99%), paraformaldehyde (90%), 2,3-dichloro-5,6-dicyano-1,4-benzoquinone (DDQ, 97%), trifluoroacetic acid (TFA, 99%), *N*-bromosuccinimide (NBS, 98%), bromophenylboronic acid, vinylphenylboronic acid, potassium carbonate (99%), and tetraphenylcyclopentadienone (TPCPD, 98%) were purchased from TCI (Tokyo, Japan). Benzaldehyde (98%), trimethylamine, pyridine (99%), [1,1′-*Bis*(diphenylphosphino)ferrocene]palladium(II) dichloride (PdCl_2_(dppf)), 98%), heptanal, bromobenzaldehyde (98%), soybean oil acrylate (SOA), *N*-methyldiethanolamine (MDEA, 99%), 4-(2-methylpropyl)phenyliodonium hexafluorophosphate (Iod), *N*-acetylcysteine (NAC, >99%), cysteamine (>98%), 5,5-dimethylpyrroline-*N*-oxide (DMPO for ESR spectroscopy), and acid red 14 (AR14, 95%) were provided from Sigma Aldrich (St. Louis, MO, USA). All the compounds were used as received. The chemical structures of the monomer, co-initiators, and porphyrin derivatives used for free-radical polymerization are described in Table 1.

Characterization. Analytical thin-layer chromatography (TLC) was performed on silica gel plates (0.25 mm) precoated with a fluorescent indicator (silica F254, VWR, Leuven, Made in Germany), and chromatograms were examined using ultraviolet light (λ = 254 nm) or an aqueous solution of KMnO_4_. Flash chromatography was performed on 40–63 μm silica gel with a mixture of solvents. NMR spectra were recorded using a Bruker Avance II 400 MHz spectrometer (Bruker BioSpin GmbH, Rheinstetten, Germany) at room temperature. Results are presented as the following: chemical shift δ (ppm), multiplicity (*s* = singlet, *d* = doublet, *t* = triplet, *q* = quartet, *quint* = quintet, *hept* = heptuplet, *m* = multiplet, *br* = broad), coupling constant J (Hz), and integration. Chemical shifts were referenced to the residual CDCl_3_ non-deuterated signal (δ^1^H = 7.26 ppm and δ^13^C = 77.16 ppm). Infrared spectra were recorded using a Bruker Tensor 27 spectrometer in ATR mode (Bruker Optics GmbH & Co. KG, Ettlingen, Germany). UV–visible absorption spectra were recorded using a PerkinElmer Lambda 2 UV–vis spectrophotometer (PerkinElmer, Waltham, MA, USA). High-resolution mass spectra were obtained from the SALSA platform from ICOA/CBM (FR2708) laboratory of the Université of Orléans by electrospray ionization using a Bruker maXis mass spectrometer (Bruker Daltonics GmbH & Co. KG, Bremen, Germany).

Synthesis of dipyrromethane. In air, a 500 mL rounded-bottomed flask equipped with a stirring bar was charged with pyrrole (130 mL, 1.87 mol, 4.0 equiv), paraformaldehyde (1.41 g, 0.47 mol, 1.0 equiv), acetic acid (200 mL), and methanol (65 mL). The flask was closed with a septum, and the reaction was stirred at room temperature for 16 h. Then, the reaction mixture was diluted with DCM (400 mL), and the resulting solution was washed with water (2 × 200 mL) and 1.0 M aq NaOH (2 × 200 mL). The organic layer was dried over Na_2_SO_4_, and the solvent and the excess pyrrole were successively removed by evaporation under reduced pressure. The residue was purified by flash chromatography (silica, DCM/EP: 5/5) to afford the expected product (1.923 g, 7%) as a white solid, which turned black under air.

^1^H NMR (400 MHz, CDCl_3_): δ 7.63 (s, 2 H, 6.61 (dd, J = 4.2, 2.6 Hz, 2 H), 6.21 (dd, J = 5.7, 2.8 Hz, 2 H), 6.09 (d, J = 0.6 Hz, 2 H), 3.94 (s, 2 H). ^13^C NMR (100 MHz, CDCl_3_): δ 129.2 (2 C), 117.4 (2 CH), 108.4 (2 CH), 106.6 (2 CH), 26.4 (CH_2_).

Synthesis of 5,15-diphenylporphyrin **(1)**. A 1 L rounded-bottomed flask equipped with a stirring bar, purged with argon, and protected from light by an aluminium foil was charged with dipyrromethane (1 g, 6.8 mmol, 1.0 equiv), benzaldehyde (0.72 mL, 7.1 mmol, 1.05 equiv), and DCM (1 L). The solution was degassed by argon bubbling for 15 min, and TFA (0.42 mL, 5.4 mmol, 0.8 equiv) was added. The reaction was stirred at room temperature for 18 h. DDQ (2.2 g, 9.52 mmol, 1.4 equiv) was then added, and the mixture was stirred at room temperature for 30 min. After addition of triethylamine (5 mL), the solvent was removed under reduced pressure, and the residue was purified by flash chromatography (silica, DCM/EP: 5/5) to afford the desired product as a purple solid (1.5 g, 39%). ^1^H NMR (400 MHz, CDCl_3_): δ (ppm) 10.33 (s, 2H), 9.41 (d, J = 4.6 Hz, 4H), 9.10 (d, J = 4.6 Hz, 4H), 8.29 (dd, J = 6.5, 2.9 Hz, 4H), 7.83–7.81 (m, 6H), −3.11 (s, 2H). ^13^C NMR (100 MHz, CDCl3): δ (ppm) 147.4 (4C), 145.4 (4C), 141.6 (2C), 135.0 (4CH), 131.8 (4CH), 131.2 (4CH), 127.9 (4CH), 127.1 (2CH), 119.3 (2C), 105.4 (2CH).

Synthesis of 5,10-diphenyl-10,20-dibromoporphyrin **(2)**. A 1 L rounded-bottom flask equipped with a stirring bar was charged with 5,15-diphenylporphyrin (1.2 g, 2.5 mmol, 1.0 equiv), DCM (870 mL), and pyridine (1.17 mL). The mixture was treated with NBS (945 mg, 5.3 mmol, 2.1 equiv) at 0 °C for 3 h and monitored by TLC [until the full conversion was reached, NBS (90 mg, 0.5 mmol, 0.2 equiv) was added every hour]. The reaction was quenched with acetone (60 mL), the solvent was removed under reduced pressure, and the residue was triturated in methanol and filtered several times to afford the desired product (1.29 g, 82%) as a purple solid. ^1^H NMR (400 MHz, CDCl_3_): δ 9.62 (d, J = 4.7 Hz, 4H), 8.84 (d, J = 4.6 Hz, 4H), 8.16 (d, J = 7.1 Hz, 4H), 7.91–7.68 (m, 6H), −2.73 (s, 2H) (Figure S1). ^13^C NMR (100 MHz, CDCl_3_): δ 141.51 (4C), 134.65 (4CH), 128.20 (2CH), 126.98 (4CH), 121.56 (2C), [16C not detected]. HRMS (ESI/Q-TOF): *m*/*z* [M + H^+^] calcd for C_32_H_21_N_4_Br_2_ 619.01127; found 619.0118.

Synthesis of 5,15-diphenyl-10,20-bis(4-bromophenyl) porphyrin **(P3)**. A 25 mL rounded-bottomed flask equipped with a stirring bar was charged with 5,10-diphenyl-10,20-dibromoporphyrin (50 mg, 0.08 mmol, 1.0 equiv), bromophenylboronic acid (35 mg, 0.176 mmol, 2.2 mmol), K_2_CO_3_ (66 mg, 0.48 mmol, 6 equiv), THF (7.2 mL), and water (0.8 mL). The reaction mixture was flushed with Ar, PdCl_2_(PPh_3_)_2_ (6 mg, 0.008 mmol, 0.1 equiv) was added, and then the reaction was heated to reflux for 16 h. The resulting solution was washed with water (2 × 10 mL) and brine (10 mL). The solvent was removed under reduced pressure, and the residue was purified by flash chromatography (silica, DCM/EP: 3/7) to afford the desired product as a purple solid (39 mg, 63%). ^1^H NMR (400 MHz, CDCl_3_): δ 8.85 (dd, J = 17.4, 4.2 Hz, 8H), 8.21 (d, J = 7.1 Hz, 4H), 8.08 (d, J = 7.9 Hz, 4H), 7.89 (d, J = 8.0 Hz, 4H), 7.80–7.75 (m, 6H), −2.82 (s, 2H). ^13^C NMR (100 MHz, CDCl_3_): δ 142.15 (2C), 142.06 (4C), 141.16 (4C), 135.98 (4CH), 135.21 (4CH), 134.67 (4CH), 130.02 (4CH), 127.97 (4CH), 126.88 (4CH), 125.21 (2CH), 122.60 (2C), 120.63 (2C), 120.55 (2C), 118.78 (2C) (Figures S2–S4). HRMS (ESI/Q-TOF): *m*/*z* [M + H^+^] calcd for C_44_H_29_N_4_Br_2_ 771.0753; found 771.0751. ν_max_ (ATR)/cm^−1^: 3319, 3040, 2704, 2336, 1564, 1470, 1340, 1169, 1063, 974, 789, 714.

Synthesis of 5,15-diphenyl-10,20-bis(4-vinylphenyl) porphyrin **(P4)**. A 25 mL rounded-bottomed flask equipped with a stirring bar was charged with 5,10-diphenyl-10,20-dibromoporphyrin (50 mg, 0.08 mmol, 1.0 equiv), vinylphenylboronic acid (26 mg, 0.176 mmol, 2.2 mmol), K_2_CO_3_ (66 mg, 0.48 mmol, 6 equiv), THF (7.2 mL), and water (0.8 mL). The reaction mixture was flushed with Ar, PdCl_2_dppf (6 mg, 0.008 mmol, 0.1 equiv) was added, and then the reaction was heated to reflux for 16 h. The resulting solution was washed with water (2 × 10 mL) and brine (10 mL). The solvent was removed under reduced pressure, and the residue was purified by flash chromatography (silica, DCM/EP: 3/7) to afford the desired product as a purple solid (30 mg, 57%).

^1^H NMR (400 MHz, CDCl_3_): δ 9.01–8.67 (m, 8H), 8.27–8.17 (m, 8H), 7.82–7.75 (m, 10H), 7.07 (dd, J = 17.6, 10.9 Hz, 2H), 6.08 (d, J = 17.6 Hz, 2H), 5.50 (d, J = 10.7 Hz, 2H), −2.77 (s, 2H). ^13^C NMR (100 MHz, CDCl_3_): δ 136.84(4 CH), 134.99 (4 CH), 134.69 (4 CH), 127.87 (4 CH), 126.84 (4 CH), 124.74 (4 CH), 114.81 (2CH), [22C not detected] (Figures S5 and S6). HRMS (ESI/Q-TOF): *m*/*z* [M + H^+^] calcd for C_48_H_35_N_4_ 667.2856; found 667.2853; *m*/*z* [M + 2H^+^] calcd for C_48_H_35_N_4_ 334.1465; found 335.1465. ν_max_ (ATR)/cm^−1^: 3319, 2947, 2704, 2355, 1458, 1256, 1080, 1013, 791, 719.

Theoretical calculation. Density functional theory (DFT) and time-dependent density functional theory (TD-DFT) calculations were carried out with the Gaussian 16 Revision B.01 package [69]. The functional B3LYP combined with the 6-31G(d) basis set was used for the optimization of the porphyrin structures and for frequency calculations. Lack of imaginary frequencies was controlled to confirm the true minima of the optimization. The TD-DFT using the same level of theory was used to compute UV–vis absorption spectra. The solvent effects were considered in the polarizable continuum model (PCM).

Irradiation source. Four light-emitting diodes (LEDs) were used for the photopolymerization experiments: LED@385 nm (35 mW·cm^2^), LED@405 nm (118 mW·cm^2^), LED@455 nm (24 mW·cm^2^), and LED@530 nm (15 mW·cm^2^). A UV lamp (Hamamatsu-LC8, 4500 mW·cm^−2^, λ = 365 nm) (Hamamatsu LC-8, Shizuoka, Japan) was used for the observation of reactive oxygen species (ROS) and the depollution experiments.

Steady state photolysis. Photolysis experiments were performed in dichloromethane, under air, and under LED@405 nm irradiation. The absorbance of **P3** and **P4** solutions with and without the presence of co-initiators (i.e., MDEA, Iod, or cysteamine) was followed upon light irradiation. [**P3**] = 1.85 × 10^−5^ M, [**P4**] = 2.9 × 10^−6^ M, [MDEA] = 2.8 × 10^−2^ M, [Iod] = 2.9 × 10^−5^ M, [cysteamine] = 1.7 × 10^−4^ M.

Steady state fluorescence measurement. Fluorescence experiments were performed using FluoroMax + spectrofluorometer from Horiba (Horiba Ltd., Kyoto, Japan). Emission spectra were obtained after the excitation of **P3** and **P4** at 649 and 646 nm, respectively.

Phosphorescence. The phosphorescence spectra were recorded using a FluoroMax-4 spectrofluorometer (Horiba Scientific, Kyoto, Japan) equipped with a Xe-pulsed lamp. Luminescence measurements were performed in a glassy matrix of 2-methyltetrahydrofuran (2-MTHF) at 77 K. The sample was placed in a 5 mm diameter quartz tube inside a Dewar filled with liquid nitrogen.

Laser Flash Photolysis. Nanosecond flash photolysis was performed using a nanosecond Nd:TAG laser (Powerlite 9010, Continuum, Santa Clara, CA, USA) operating at 5 Hz with 7–8 ns impulsion time and working at 385 nm, as previously described [70]. Measurements were performed under a saturated argon atmosphere at room temperature.

Electronic Paramagnetic Resonance (EPR). The EPR spectra were recorded in situ upon/after a defined exposure as described previously in ref. [12,70]. The solutions were prepared in chloroform (for spectroscopy Uvasol^®^, Merck, Darmstadt, Germany) and carefully saturated with argon were irradiated at 295 K directly in the EPR resonator using a LED@400 nm source (λ_max_ = 400 nm; Bluepoint LED, Hönle UV Technology, Türkenfeld, Germany). The 5,5-dimethyl-1-pyrroline N-oxide (DMPO, Sigma-Aldrich, distilled and stored at –20 °C before the application) was applied as the spin trapping agent. The X-band cw-EPR spectra (modulation frequency of 100 kHz) were monitored using the EMX*plus* spectrometer (Bruker) equipped with the high-sensitivity probe head (Bruker) in a small quartz flat cell (Wilmad-LabGlass, WG 808-Q, Vineland, NJ, USA). The *g*-factors were determined with an uncertainty of ±0.0001 exploiting a nuclear magnetic resonance teslameter (ER 036TM, Bruker) and an integrated frequency counter. The experimental EPR spectra were analyzed by the WinEPR acquisition software (Bruker Biospin GmbH, version 4.3, Billerica, MA, USA), and the calculations of spin-Hamiltonian parameters and relative concentrations of individual DMPO adducts were performed with the EasySpin toolbox working on MatLab^®^ platform (version 6.0.11). The standard EPR spectrometer settings: microwave frequency, ~9.43 GHz; microwave power, 10.80 mW; center field, ~336.0 mT; sweep width, 4–10 mT; gain, 2.00 × 10^5^; modulation amplitude, 0.025–0.1 mT; sweep time, 45 s; time constant, 10.24 ms; number of scans, 5–10.

Cyclic voltammetry. The cyclic voltammograms of **P3** and **P4** were obtained using an AUTOLAB potentiometer/galvanometer employing GPES electrochemical software 4.9 (Utrecht, The Netherlands). A three-electrode cell configuration was employed, with a glassy electrode as the working electrode, a saturated calomel electrode (SCE) as the reference one, and a gold wire electrode as the counter electrode. The DMF solution of *n*Bu_4_NBF_4_ (0.1 M) was used as a supporting electrolyte.

Kinetic studies. Photosensitive formulations were laid on a BaF_2_ pellet (thickness of the layer = 12 μm) and irradiated with various LEDs in a laminate or under air. The absorbance decrease in the SOA acrylate function was followed by real-time Fourier transform infrared spectroscopy (RT-FTIR, JASCO FTIR 4700) at 1636 cm^−1^, thus allowing us to determine the final acrylate conversions and polymerization rate.

Coating preparation. The photosensitive formulations, i.e., **P3**/MDEA/SOA and **P4**/MDEA/SOA (0.5 wt% porphyrin and 5 wt% MDEA in SOA), were first deposited on a glass substrate, which was freshly cleaned with acetone and ethanol. Formulations were then irradiated for 10 min under LED@405 nm in a laminate.

Detection of singlet oxygen. A solution of TPCPD in methanol (3.4 × 10^−4^ M) was used to investigate the photogeneration of singlet oxygen. Materials are embedded in 30 mL of TPCPD solution and irradiated with a UV light source (*Hamamatsu-LC8*, 4500 mW·cm^−2^, λ = 365 nm). The singlet oxygen formation was followed by a decrease in the absorbance of TPCPD at 510 nm.

Dye degradation experiment. A pellet containing porphyrin was immersed in 30 mL of an aqueous solution of AR14 with an initial concentration of 10 μM and irradiated under UV light (Hamamatsu-LC8, λ = 365 nm). Experiments were carried out in dynamic mode, i.e., the sample was continuously under stirring to ensure a constant supply of AR14 dye on the surface of the pellet. This dynamic fluid regime can also maximize the removal of the formed reaction products from the catalyst surface [63]. It is worth noting that the distance between the UV lamp and the sample surface was maintained at 10 cm in all measurements, and the UV irradiation power received at the sample surface was 35 mW∙cm^−2^. The photocatalysis reaction was monitored by UV–visible spectrophotometry (Lambda 35, Perkin Elmer), measured by using a sample of the solution taken every 15 min for 5 h, allowing then to determine the photodegradation rate of the pollutant in the water. In order to compare the photodegradation efficiency of our innovative system according to the different experimental conditions, the degradation rate (*X*%) in the aqueous solution was determined using Equation (1).

(1) $$X(\%)=\frac{A_{0}-A_{t}}{A_{0}}\times 100$$

where $A_{0}$ and $A_{t}$ stand, respectively, for the maximal absorbance peak, i.e., λ = 515 nm for AR14 before illumination (t = 0 s), and at time t during the irradiation process.

In order to highlight the photocatalytic properties of the synthesized porphyrin-based materials, a photolysis test, without any sample, was carried out. Furthermore, an adsorption test without UV light was also performed to determine if the absorbance decrease in the pollutant was only due to the adsorption of AR14 on the surface of materials.

## 3. Results and Discussion

### 3.1. Synthesis of Porphyrin Derivatives

**Figure 1:**
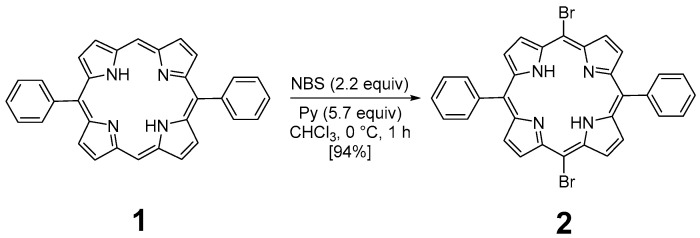
Dibromation of 5,15-diphenylporphyrin **1**.

The preparation of **P3** and **P4** required the synthesis of *meso*-substituted A_2_B_2_-*trans*-porphyrins. In that way, we focused our attention on the use of the Suzuki–Miyaura cross-coupling reaction between diphenyl-dibromo-*trans*-porphyrin **2** and arylboronic acids [71,72]. Indeed, the intermediate molecule **2** could be easily obtained in a 94% yield by dibromation of 5,15-diphenylporphyrin **1** [73] with *N*-bromosuccinimide (NBS) in chloroform in the presence of pyridine (Figure 1) [74,75].

**Figure 2:**
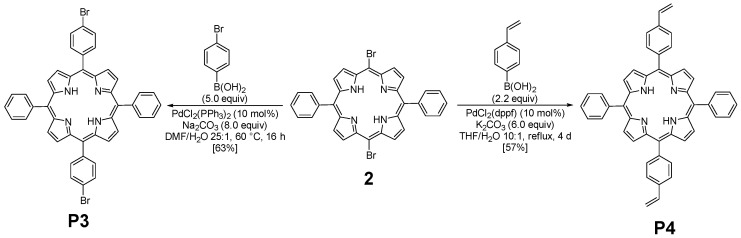
Synthesis of **P3** and **P4**.

The functionalization of **2** with arylboronic acids could be efficiently achieved using palladium catalysis (Figure 2). Indeed, the use of standard conditions (10 mol% of PdCl_2_(PPh_3_)_2_ with 8.0 equiv of Na_2_CO_3_) allowed the double Suzuki–Miyaura cross-coupling of **2** with 4-bromoboronic acid. The use of an excess of this last reagent was essential to ensure the introduction of the two aryl groups, leading to the isolation of **P3** with a yield of 63%. Even if these conditions were revealed to be less efficient with 4-vinylphenylboronic acid (30% isolated yield), a slight modification of the reaction conditions was necessary. Indeed, PdCl_2_(dppf) was used as a catalyst, and **P4** was obtained with a 57% yield. The structures of compounds **P3** and **P4** were confirmed using HRMS and NMR analyses. ^1^H NMR (Figures S2 and S5) was particularly insightful. The comparison of **P3** and **P4** NMR signals with the reference one (molecule 2) revealed the presence of characteristic peaks. Specifically, the COSY ^1^H-^1^H NMR spectrum (Figure S3) of **P3** displays several interesting correlations: one between the two doublets around 8.85 ppm, likely attributed to the protons of the pyrrole rings; another one between signals at 8.21 and 7.80, corresponding to the protons of the phenyl rings; more importantly, a cross-signal between two additional doublets at 8.08 and 7.89 ppm, each integrating for four protons, indicating the presence of a *para*-disubstituted phenyl ring. In contrast, the presence of vinyl groups in **P4** was readily confirmed by the three corresponding signals observed at 7.07, 6.08, and 5.50 ppm.

### 3.2. Computational Study and Absorbance Properties of Both Porphyrins

**Figure 3:**
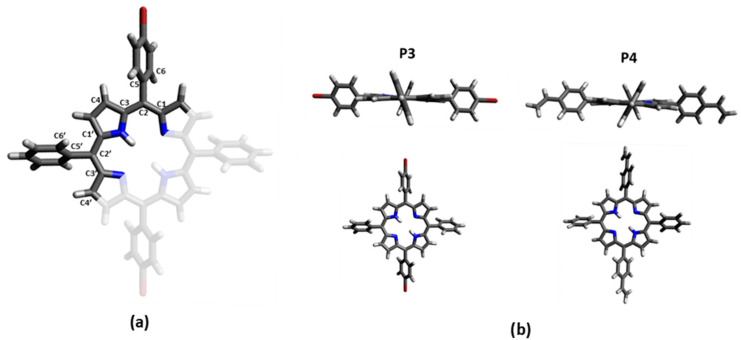
(**a**) Labelling of carbons in used porphyrin. Red corresponds to the substituted part of the porphyrin, corresponding to the brome for **P3** and the vinyl group for **P4**. (**b**) Molecular geometry of the ground state of **P3** and **P4** optimized in DCM at the B3LYP level using 6-31G(d) basis set.

The optimized geometry of **P3** and **P4** in the ground state (see Tables S3 and S4) and in the first excited state in DCM have been computed using DFT calculations at the B3LYP level using 6-31G(d) basis set to explore the influence of the substituents on the porphyrin core. The optimized structures in the ground state reveal a relatively planar saddle configuration for the porphyrin core (the C1-C2-C3-C4 and C1′-C2′-C3′-C4′ dihedral angles are −176.2° and 175° for **P3**, and 174.7° and −176° for **P4**; Figure 3). The phenyl groups are twisted compared to the porphyrin plane by a C1′-C2′-C5′-C6′ dihedral angle of −65.6° for **P3**, and 65.5° for **P4**. The substituted phenyl groups follow the same trend, with a C1-C2-C5-C6 dihedral angle a little more accentuated: 65.4° for the bromo-phenyl of **P3** and −63.6° for the vinyl-phenyl of **P4**.

**Figure 4:**
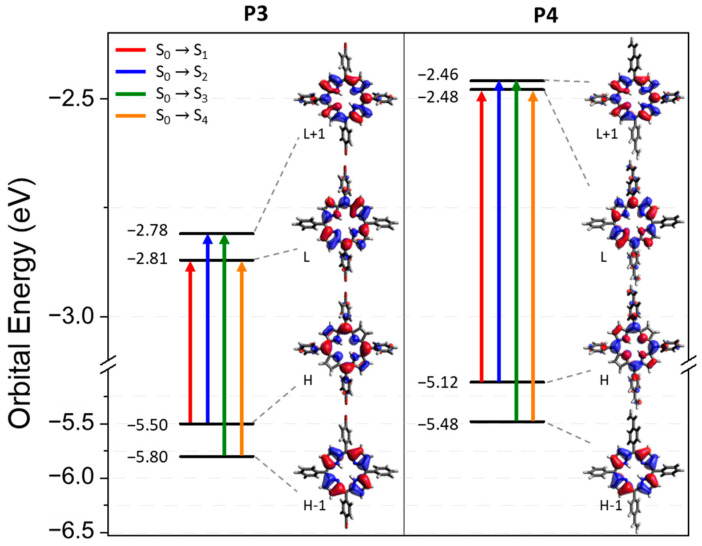
Energy levels and representation of the LUMO+1, LUMO, HOMO, and HOMO-1 orbitals for **P3** and **P4** from a B3LYP/6-31G(d) calculation on the ground state optimized geometry. The biggest contribution in the TD-DFT transitions from the ground state to the first four excited states is highlighted in color. Contributions are given in Table S1 in the Supplementary Information.

**Figure 5:**
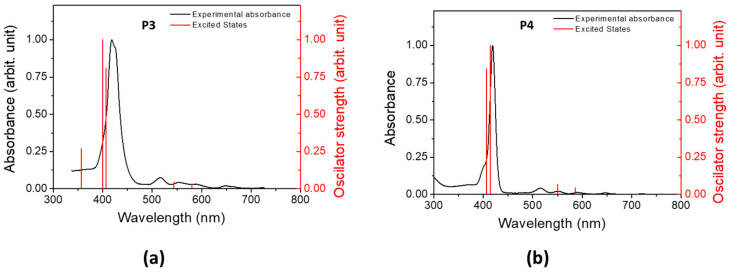
Experimental absorption (black) spectra in DCM and computed excited states (red) of (**a**) **P3** and (**b**) **P4**.

The electron-density distribution of the four frontier molecular orbitals (MOs) of **P3** and **P4,** with their associated energies, is displayed in Figure 4. For both porphyrins, LUMO and LUMO+1 are very close in energy. The electronic distributions of the different MOs are similar for **P3** and **P4** and are mainly located in the porphyrin core. This could explain the slight difference between the absorption spectra of **P3** and **P4**. The calculated electronic transitions (Figure 5 and Table S1) on static structures give qualitative agreement with the experimental data. Indeed, the calculated energies associated with their oscillation strengths (*f*) reveal two low-intensity transitions, located at around 600 nm, corresponding to the Q bands (without their vibronic overtones), as well as two high-intensity transitions, which correspond to the Soret band at around 420 nm.

### 3.3. Experimental Absorbance and Fluorescence Spectra

**Figure 6:**
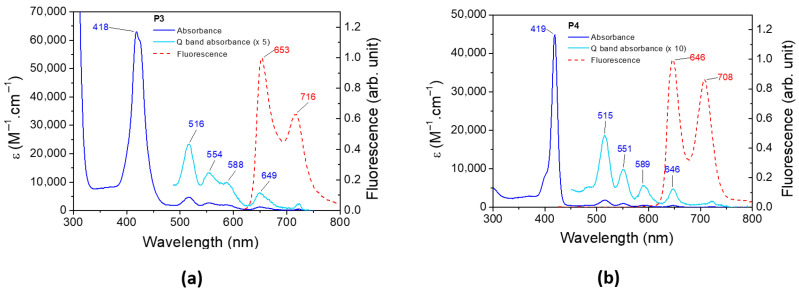
Experimental absorbance and fluorescence spectra of (**a**) **P3** and (**b**) **P4** in DCM.

The UV–visible spectrum and the normalized emission spectrum of **P3** and **P4** are illustrated in Figure 6. The absorbance, emission, and phosphorescence properties of both porphyrins are depicted in Table 2. The absorbance spectra of **P3** and **P4** present a classical π–π* transition [76]. The Soret band at 418 nm and 419 nm for **P3** and **P4**, respectively, corresponds to the S_0_ → S_3_ and S_0_ → S_4_ transitions. Q bands are assigned to the S_0_ → S_1_ and S_0_ → S_2_ transitions, and their corresponding vibronic overtones are localized between 500 and 600 nm. The fluorescence spectrum of **P3** (and **P4**) presents two bands, one corresponding to the S_1_ → S_0_ transition and a second band at a longer wavelength resulting from the relaxation of S_1_ to a vibronic state of the fundamental state. The bands are separated by 1347 cm^−1^ and 1355 cm^−1^ for **P3** and **P4**, respectively, which are in full agreement with the literature [77]. The phosphorescence spectra of **P3** and **P4** are displayed in Figure S7.

**P3** and **P4** were used as part of new photoinitiating systems when combined with co-initiators, i.e., MDEA, Iod, cysteamine, or *N*-acetylcysteine (NAC), to promote the FRP of SOA. Prior to RT-FTIR experiments, the photochemical properties of the porphyrin-based photoinitiating systems are described in detail by steady-state photolysis, fluorescence, laser flash photolysis, and EPR-ST experiments. All these properties are summarized in Table S2, including the redox properties (E_ox_ and E_red_) of both porphyrins (Figure S8), and the energies of the excited singlet (E_S_) and triplet states (E_T_) determined by fluorescence and phosphorescence, respectively. These results were used to determine the free-energy changes (ΔG_S_ and ΔG_T_ at the singlet and triplet excited states, respectively) according to the Rehm–Weller equation (Equation (S1)) and given in Table 2, thus confirming that electron transfer reactions may be favorable or not.

### 3.4. Reactivity of **P3** and **P4**-Based Photoinitiating Systems Under Light Irradiation

**P3** and **P4** were used as part of new photoinitiating systems when combined with co-initiators, i.e., MDEA, Iod, cysteamine, or *N*-acetylcysteine, to promote the FRP of SOA. Prior to RT-FTIR experiments, the photochemical properties of the porphyrin-based photoinitiating systems are described in detail by steady-state photolysis, fluorescence, laser flash photolysis, and EPR-ST experiments. All these properties are summarized in Table S2, including the redox properties (E_ox_ and E_red_) of both porphyrins (Figure S8), and the energies of the excited singlet (E_S_) and triplet states (E_T_) determined by fluorescence and phosphorescence, respectively. These results were used to determine the free-energy changes (ΔG_S_ and ΔG_T_ at the singlet and triplet excited states, respectively) according to the Rehm–Weller equation (Equation (S1)) and given in Table 3, thus confirming that electron transfer reactions may be favorable or not.

When used alone, and under LED@405 nm irradiation, the absorbance of both porphyrins does not change, demonstrating their high stability under light exposure (Figure S9). However, the irradiation of **P3** and **P4** in the presence of DMPO leads to a superposition of individual signals of DMPO adducts generated via the reaction of DMPO with free radicals. The spin-Hamiltonian parameters of corresponding DMPO adducts elucidated from the simulations are as follows: (i) •DMPO-CHCl_2_ (*a*_N_ = 1.401 mT, *a*_H_^β^ = 1.997 mT; *g* = 2.0061), (ii) •DMPO-CCl_3_ (*a*_N_ = 1.363 mT, *a*_H_^β^ = 1.585 mT; *g* = 2.0062), and (iii) •DMPO-CR (*a*_N_ = 1.483 mT, *a*_H_^β^ = 2.157 mT; *g* = 2.0059), assigned to a DMPO adduct of carbon-centered radical produced most probably from porphyrins (Figure S10).

Transient absorption spectra of **P3** and **P4** are determined by laser flash photolysis (LFP) between 350 and 750 nm under laser excitation at λ = 385 nm (Figure S11A,B). The absorption maxima corresponding to the triplet excited states of **P3** and **P4** are observed at 444 nm and 460 nm, respectively. As expected, the triplet states of both porphyrins are highly sensitive to oxygen, resulting in a tremendous decrease in their lifetime, going from τ = 25.7 μs to τ = 0.95 μs, and 28.2 μs to 0.63 μs for **P3** and **P4**, respectively (Figure S11C,D).

#### 3.4.1. Effect of the Addition of MDEA

**Figure 7:**
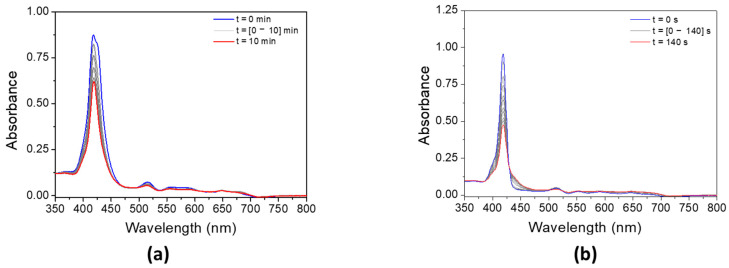
Steady-state photolysis of a DCM solution of (**a**) **P3** and (**b**) **P4** in the presence of MDEA after LED@405 nm irradiation. [**P3**] = 1.9 × 10^−5^ M, [**P4**] = 2.9 × 10^−6^ M, [MDEA] = 2.8 × 10^−2^ M.

The addition of MDEA significantly decreases the absorbance of both porphyrins under LED@405 nm (Figure 7). The absorbance of **P3** is divided by 3 in 10 min, while the absorbance of **P4** decreases by half in only 140 s. These results are in good agreement with a possible electron transfer reaction between the porphyrins and MDEA. According to Table 3, the calculation of the electron-transfer Gibbs energy ΔG between **P3** in its singlet state and MDEA in its ground state is thermodynamically unfavorable (ΔG_S_^MDEA^(**P3**) = 0.04 eV > 0), contrary to **P4** (ΔG_S_^MDEA^(**P4**) = −0.25 eV < 0). Experimental investigation of the singlet state upon the addition of MDEA by fluorescence is consistent with these low ΔG values. Indeed, both porphyrins exhibit a low quenching rate of their singlet excited state in the presence of MDEA (Figure S12), with fluorescence quenching rates of K_SV_^MDEA^ (**P3**) = 9.8 M^−1^ and K_SV_^MDEA^ (**P4**) = 0.9 M^−1^.

Interestingly, the addition of MDEA to both porphyrin solutions leads to an increase in their triplet lifetime (from 25 µs to 33 µs for **P3** and from 23 µs to 49 µs for **P4**), highlighting the formation of new species (Figure S13). The electron-transfer Gibbs energies ΔG (ΔG_T_^MDEA^(**P3**) = 0.51 eV and ΔG_T_^MDEA^ (**P4**) = 0.23 eV) are unfavorable for an electron transfer reaction between MDEA and the triplet excited state of porphyrins. Nevertheless, the irradiation of both porphyrins at λ = 405 nm in the presence of MDEA and DMPO shows some individual DMPO adducts: with a dominant signal of •DMPO-C(α-aminoalkyl) characterized by the spin-Hamiltonian parameters *a*_N_ = 1.509 mT, *a*_H_^β^ = 1.730 mT, *a*_H_^γ^ = 0.183 mT, *a*_H_^γ^ = 0.0730 mT; *g* = 2.0057 [78,79] and two less-abundant signals of DMPO adduct with a carbon-centered radical (*a*_N_ = 1.500 mT, *a*_H_^β^ = 2.164 mT; *g* = 2.0059) and •DMPO-CHCl_2_ reflecting interaction of photoinitiating system with solvent (Figure S14).

#### 3.4.2. Effect of the Addition of Iod

**Figure 8:**
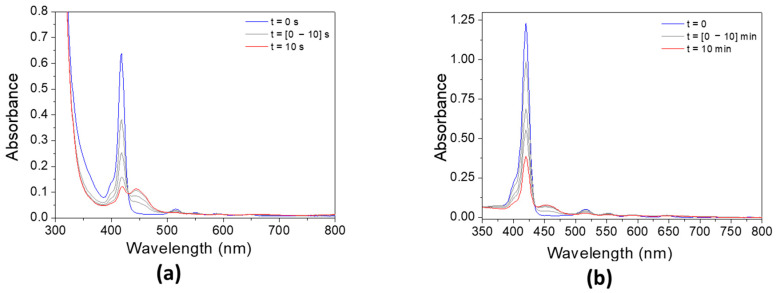
Steady-state photolysis of (**a**) **P3**/Iod and (**b**) **P4**/Iod under air after LED @405 nm exposure. [**P3**] = 1.9 × 10^−5^ M, [**P4**] = 2.9 × 10^−6^ M, [Iod] = 2.9 × 10^−5^ M. Solvent = DCM.

The addition of Iod has a strong effect on the absorbance of both porphyrins (Figure 8). The absorbance of **P3** significantly decreases after only 10 s, while the photolysis rate of **P4**/Iod photoinitiating system remains slower than that observed with **P3**. Interestingly, and concomitantly to the decrease in the absorbance of both porphyrins, an increase in the absorbance can be observed at 450 nm, thus indicating the formation of new absorbing products.

The singlet state study of the porphyrins revealed a quenching upon the addition of Iod (Figure S15). Indeed, the Stern–Volmer constants are relatively high (K_SV_^Iod^ (**P3**) = 2600 M^−1^ and K_SV_^Iod^ (**P4**) = 1756 M^−1^), indicating a rapid quenching between porphyrins and Iod. These results are consistent with the low values of free-energy change ΔG, corresponding to a favorable electron transfer reaction: ΔG_S_^Iod^(**P3**) = −0.11 eV and ΔG_S_^Iod^ (**P4**) = −0.34 eV. At the triplet excited state, the Rehm–Weller equation predicts a lower reactivity (ΔG_T_^Iod^(**P3**) = +0.28 eV and ΔG_T_^Iod^ (**P4**) = +0.07 eV), which is consistent with the low interaction observed using LFP (Figure S16). EPR spectra obtained upon irradiation of **P3** and **P4** in the presence of Iod in CHCl_3_ and under argon present a broad signal with unresolved hyperfine structures (Figure S17), which can be assigned to the corresponding porphyrin radical cations, **P3**^•+^ and **P4**^•+^ (*g* = 2.0040 and *g* = 2.0032 for **P3**/Iod and **P4**/Iod, respectively). These results confirm an electron transfer reaction at the singlet excited state of **P3** (or **P4**) and Iod.

#### 3.4.3. Effect of the Addition of Cysteamine

The addition of cysteamine has no significant influence on the evolution of the porphyrin absorbance after 10 min of irradiation (Figure S18). However, despite a low favorable free-energy change at the singlet state (ΔG_S_^cysteamine^(**P3**) = 0.24 eV and ΔG_S_^cysteamine^ (**P4**) = −0.05 eV), the study of the singlet state shows an interaction between porphyrins and cysteamine, with a high quenching constant of K_SV_^cysteamine^ (**P3**) = 235 M^−1^ and K_SV_^cysteamine^ (**P4**) = 351 M^−1^ for **P3** and **P4**, respectively (Figure S19). The EPR spectra confirmed this phenomenon by the formation of a signal of limited stability, typical for ^•^DMPO-SR spin adduct (*a*_N_ = 1.369 mT, *a*_H_^β^ = 1.247 mT, *a*_H_^γ^ = 0.094 mT, *a*_H_^γ^ = 0.082 mT; *g* = 2.0059 [12,79,80] when irradiated with DMPO (Figure S20). The study of the interaction between cysteamine and porphyrins at the triplet state reveals a low modification for **P4**, and a small increase in the lifetime of the excited state of **P3** (Figure S21). These results indicate that the photoinduced electron transfer reaction between the excited porphyrins and cysteamine is closely associated with the formation of porphyrin radical anions.

#### 3.4.4. Effect of the Addition of N-Acetylcysteine (NAC)

The effects of the addition of NAC on the photochemical properties of porphyrins at the ground state are less pronounced than other co-initiators. Steady-state photolysis (Figure S22) does not reveal any interaction, since the absorbance of both porphyrins remains stable after 10 min of irradiation, without the appearance of new peaks. The study of the singlet excited state reveals fluorescence quenching upon gradual addition of NAC to a **P3** solution (Figure S23), with a Stern–Volmer constant of K_SV_**^P3^**^/NAC^ = 216 M^−1^. On the contrary, the addition of NAC to a solution of **P4** results in an increase in fluorescence intensity, suggesting the presence of new species. In the triplet excited state, the addition of NAC to porphyrin-based solutions has no quenching effect, but an increase in the lifetime of the excited species is observed for **P3** (Figure S24). Indeed, it can be observed that the decay traces require a longer time to reach zero, implying the presence of a more stable porphyrin excited state. On the other hand, no significant change is observed for **P4**. The EPR-ST experiments revealed the presence of a DMPO-SR adduct upon irradiation of porphyrin solutions in the presence of NAC and BMPO, consistent with the presence of a thiyl radical [79] (Figure S25). The Hamiltonian parameters of this adduct are *a*_N_ = 1.417 mT, *a*_H_^β^ = 1.409 mT, *a*_H_^γ^ = 0.086 mT, *a*_H_^γ^ = 0.063 mT, and *g* = 2.0061. It should be noted that this adduct is also present without irradiation, which can be explained by the formation of a Forrester–Hepburn mechanism (Figure S26), but this adduct is present in much smaller quantities.

### 3.5. Free-Radical Polymerization of SOA

**Figure 9:**
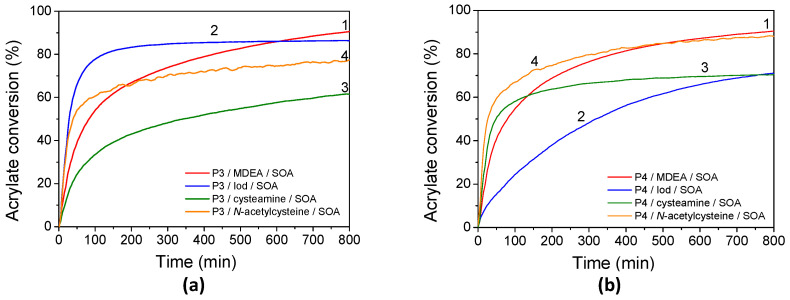
Kinetic profiles of FRP of SOA in laminate for (**a**) **P3** and (**b**) **P4** in the presence of (1) MDEA, (2) Iod, (3) cysteamine, and (4) N-acetylcysteine under LED@405 nm exposure.

FRP has been carried out using a porphyrin in combination with MDEA, Iod, cysteamine, or NAC and using SOA as an acrylate monomer. Kinetic profiles of SOA under LED@405 nm irradiation under laminate and under air are displayed in Figure 9, the final acrylate monomer conversions after 800 s are summarized in Table 4, and the rates of SOA polymerization are displayed in Table S5. As previously described, the addition of a co-initiator to porphyrins led to the formation of radicals, enabling the initiation of the FRP of SOA. Interestingly, high final acrylate conversions of SOA are observed using porphyrin and MDEA at different irradiation wavelengths. Maximal acrylate conversions are obtained at LED@405 nm irradiation under laminate, i.e., 91% for both porphyrins, corresponding to their maximal absorbance, and 93% and 80% for **P3** and **P4**, respectively, under air. When MDEA is employed as a co-initiator, light exposure triggers an electron transfer from the amine to the excited states of **P3** (or **P4**), followed by a proton transfer. This process generates aminoalkyl radicals and porphyrin radical anions, as confirmed by EPR-ST analysis. The photoinduced electron transfer between **P4** (or **P3**) in its singlet state and MDEA is more or less thermodynamically favorable, as indicated by the Rehm–Weller equation (ΔG_S_ **P4**/MDEA = –0.25 eV). Also, the high fluorescence quenching rates of porphyrin-based photosensitizers by MDEA, i.e., K_SV_^MDEA^ (**P3**) = 9.8 M^−1^ and K_SV_^MDEA^ (**P4**) = 0.9 M^−1^, confirm the high reactivity of the **P3** (or **P4**)/MDEA photoinitiating systems. On the contrary, the reaction from the triplet state of porphyrin-based photosensitizers appears less favorable due to the higher ΔG_T_ **P3** (or **P4**)/MDEA values compared to that from their singlet state (ΔG_T_ **P3**/MDEA = +0.51 eV and ΔG_T_ **P4**/MDEA = +0.23 eV). Under air, aminoalkyl radicals—acting as oxygen scavengers—can react with oxygen to form peroxyl radicals, which may then abstract hydrogen from nearby amino groups, regenerating the aminoalkyl radicals.

In the same conditions, CQ only affords 56% and 38% of acrylate conversion under laminate and oxygen, respectively. Upon higher irradiation wavelength, **P3** (or **P4**)/MDEA allows polymerization around 60% and 40% for both porphyrin upon 455 nm irradiation under laminate and under air, respectively. At 530 nm, SOA acrylate final conversion still reaches around 45% under laminate and 26% under air.

Kinetic profiles for FRP of SOA in the presence of **P3** (or **P4**) combined with Iod are shown in Figure 9. Acrylate conversions after 800s of UV and visible-light exposure are summarized in Table 4 and compared with benchmark systems using CQ/Iod. Polymerization proceeded rapidly under both **P3**/Iod and **P4**/Iod upon irradiation, with no detectable induction period, even when performed in air. In laminated samples under LED@405 nm, the final conversion (FC) values reached up to 86% for **P3** and 70% for **P4**. Notably, both systems also maintained high FCs, up to 70%, when irradiated at 455 and 530 nm. The overall efficiency of the porphyrin-based systems with Iod could be likely explained by the high reactivity of the phenyl radicals towards the acrylate functions and generated from the photolysis of **P3** (or **P4**)/Iod systems under light irradiation. The high reactivity of the phenyl radicals toward acrylate double bonds is also highlighted by the higher values of the rates of SOA polymerization with porphyrins/Iod/SOA formulations compared with porphyrins/MDEA/SOA ones. The rates of SOA polymerization using Iod are twice as high as observed with MDEA-based formulations (Table S5). However, porphyrin-based systems with Iod appeared to show high sensitivity to oxygen compared to those with MDEA. This is highlighted by the lower final acrylate conversion of SOA with polymerization under air compared to the one under laminate for the Iod-based systems. It is well known that aminoalkyl radicals (MDEA•) can act as an oxygen scavenger [1] according to the following equations (Equations (2) and (3)). Under air, the aminoalkyl radicals react with oxygen to form peroxyl-based radicals, which are able to abstract hydrogen from MDEA and regenerate the aminoalkyl radicals.

MDEA• + O_2_ → MDEA-OO• (2)

MDEA-OO• + MDEA → MDEA-OOH + MDEA•(3)

These results suggest that **P3** (or **P4**)/Iod photoinitiating systems are more likely to react from their triplet excited state.

These Iod-based photoinitiating systems are particularly interesting, as FRP of SOA could be performed from the singlet excited states; indeed, the Stern–Volmer constants are relatively high (K_SV_^Iod^ (**P3**) = 2600 M^−1^ and K_SV_^Iod^ (**P4**) = 1756 M^−1^) and the values of the free-energy changes (ΔG_S_^Iod^(**P3**) = −0.11 eV and ΔG_S_^Iod^ (**P4**) = −0.34 eV) highlight a favorable electron transfer reaction.

To enhance the biobased content of the photopolymerizable formulations and reduce their toxicity, NAC or cysteamine was explored as a biobased FRP co-initiator for replacing the more commonly used MDEA or Iod ones. The photoinduced polymerizations of SOA in the presence of **P3** (or **P4**)/NAC and **P3** (or **P4**)/cysteamine were monitored kinetically, with the results shown in Figure 9. Monomer conversions for NAC-containing systems are particularly high when polymerized at 405 nm under air, reaching 96% and 85% for **P3** and **P4**, respectively. A similar outcome had been reported for cysteamine-based systems. The FCs of SOA with **P3**/cysteamine and **P4**/cysteamine reach 61 and 70%, respectively, in a laminate without any influence of oxygen. Interestingly, the rates of SOA polymerization (with thiol-derivatives, NAC, and cysteamine) are weakly influenced by the addition of oxygen during the polymerization (Table S5). This finding is not surprising as the photoinduced thiol-acrylate polymerizations are generally known for their rapid reaction rates and high conversions under mild conditions [81,82]. Furthermore, thiols derived from mercaptopropionate esters, such as NAC, have been described as particularly reactive toward certain alkenes [83]. Notably, systems containing 5% of cysteamine were found to polymerize at room temperature in the absence of light. To achieve optimum conditions, the cysteamine concentration had to be reduced to 1%. This can explain the low FCs of 61 and 70% for **P3** and **P4**, respectively, under 405 nm irradiation in the laminate. In this study, the addition of NAC or cysteamine leads, according to the EPR-ST results, to the formation of thiyl radicals, which are expected to add to acrylate functions. Also, and according to the free-energy changes, reactions with NAC or cysteamine may occur at the singlet excited states. Surprisingly, low or no polymerization occurred when reactions proceeded at λ > 405 nm. These unexpected results may partly stem from the extremely fast termination reactions typical of thiol-acrylate photopolymerizations [82], coupled with the fact that NAC or cysteamine are monofunctional thiols. We can also expect a combination reaction between thiyl radicals at λ > 405 nm.

### 3.6. Formation of ROS Under UV Light

**Figure 10:**
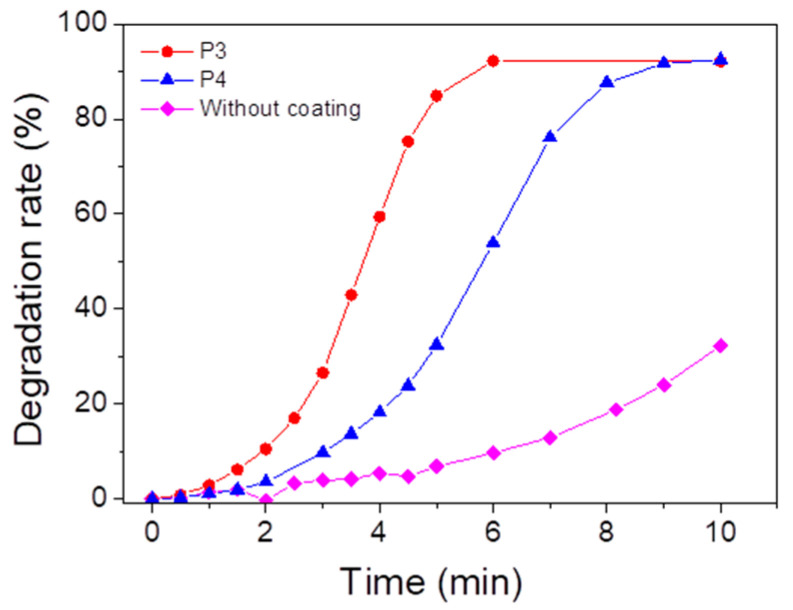
Degradation of TPCPD at 510 nm, indicating formation of singlet oxygen in the presence of **P3** or **P4** coating under UV lamp (λ_maximum_ = 365 nm) in an open-air system. [TPCPD] = 3.4 × 10^−4^ M in methanol.

In order to anticipate the depollution capability of the porphyrin-based materials, ROS formation tests have been carried out (Figure 10). The formation of singlet oxygen due to the energy transfer reaction between porphyrin and oxygen under light irradiation is monitored with the tetraphenylcyclopentadienone (TPCPD) probe [84] (the mechanism is described in Figure S27) by UV–vis spectroscopy. After 10 min of irradiation, a 32% decrease in the TPCPD absorbance is observed without the addition of material, while the addition of **P3**- and **P4**-based materials allows a decrease of 92%. We can therefore conclude that both **P3** (or **P4**)-based materials can form singlet oxygen under UV light irradiation.

### 3.7. Photo-Oxidation of AR14

**Figure 11:**
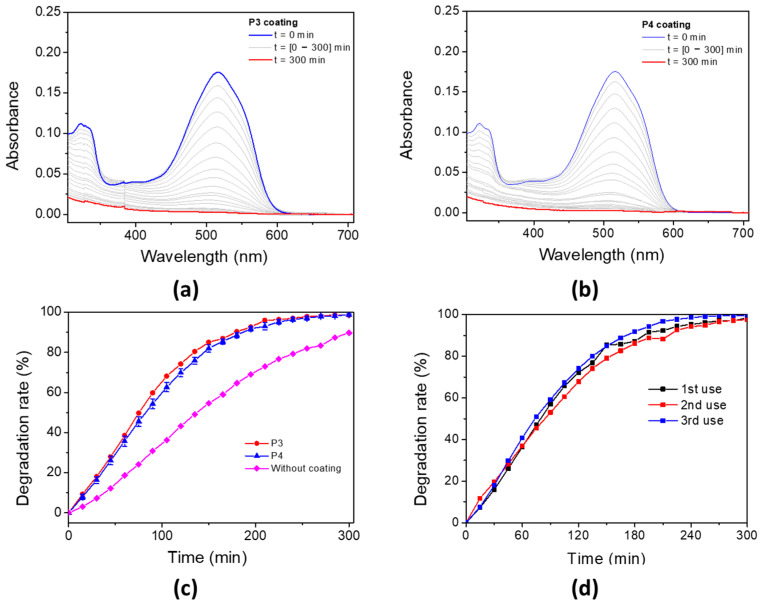
Photodegradation rate of AR14 under UV irradiation (UV lamp, λ_maximum_ = 365 nm) in an open-air system for (**a**) **P3** and (**b**) **P4** coatings. (**c**) Associated degradation rate of AR14 for **P3** coating, **P4** coating, and without coating. (**d**) **P4** coating after three uses.

The photo-oxidation capability of these porphyrin-based materials for water decontamination was studied. The porphyrin-based coatings were immersed in an aqueous solution of AR14 and irradiated under a UV lamp with a maximum emission wavelength at 365 nm. The oxidation properties of the **P3** and **P4**-based bulk materials have been evaluated by following the UV–vis absorbance of AR14 every 15 min, until its total oxidation was achieved in solution (Figure 11a for **P3** coating and Figure 11b for **P4** coating). The degradation of AR14 as a function of the irradiation time (with and without porphyrin-based materials) is described in Figure 11c. Interestingly, the addition of porphyrin-based coatings in AR14 solution accelerates its degradation under light exposure. AR14 is fully oxidized after 5 h with the presence of **P3** (or **P4**)-based coatings, whereas more than 10 h is needed for its full oxidation without coatings. It is also interesting to notice that the decrease in UV–vis absorbance of AR14 in solution and consequently its degradation is not due to its adsorption on the surface of the porphyrin-based materials but rather to its photo-oxidation by singlet oxygen under light exposure (Figure S28). Nevertheless, it is also interesting to highlight that despite the non-nanostructured **P4**-based materials, unlike TiO2 NPs or ZnO nanowires, the photocatalytic efficiency of **P4**-based materials remains remarkable. Under the same conditions (in the same laboratory, but using ZnO nanowire arrays), AR14 reached 100% degradation in 3 h with ZnO nanowires [64]. The reusability of the **P4**-based materials for the photodegradation of AR14 has also been demonstrated (Figure 11d). Interestingly, the efficiency of the porphyrin-based materials remains the same even after three cycles of irradiation, and AR14 is fully degraded after 5 h of irradiation. Regarding the reusability of the porphyrin-based material, UV–vis experiments have been performed on the materials before irradiation and after some photocatalytic process. After the third photocatalysis cycling, the absorbance of the materials changes: the Soret band and the Q bands of the porphyrin are no longer visible, but a new absorbance band at around 450 nm appears (Figure S29). However, no change was observed macroscopically at the surface of the porphyrin-based material. Therefore, the photocatalytic experiments likely lead to the formation of a secondary product without any changes in the photocatalytic efficiency. To elucidate the nature of the formation of this new product, the photolysis of **P4** in DCM solution was observed (Figure S30), and a new absorbance band appeared at 450 nm. This new species is probably associated with a tetrapyrrolic compound [85], i.e., chlorine or bacteriochlorine, which is also efficient in producing ROS under light irradiation. This could explain the photocatalytic efficiency of the **P4**-based material even after three cycling processes.

## 4. Conclusions

Two new free-metal-based porphyrins have been designed and used as a photosensitizer for initiating the free-radical photopolymerization of an acrylate bio-based monomer and as photocatalysts for water depollution. The kinetic profiles of SOA highlight the high photoinitiating properties of the porphyrin-based photoinitiating systems involving MDEA (Iod, NAC, or cysteamine). Indeed, high final acrylate conversions (> 70%) were obtained when using co-initiators under LED@405 nm irradiation and at least 50% under LED@530 nm. Interestingly, **P3** (or **P4**)/NAC systems demonstrate exceptional photoinitiating properties when FRP of SOA occurs at 405 nm under air, reaching 96% and 85% for **P3** and **P4**, respectively. These results are fully explained by the formation of efficient initiating radical species (carbon-centered thiyl radicals) through electron transfer process or H abstraction reactions between the singlet or triplet excited states of **P3** (or **P4**) and the ground state of the co-initiators. The final polymer/porphyrin-based materials obtained and based on SOA demonstrated very interesting properties for the photo-oxidation of AR14 in water, under light irradiation. Interestingly, the addition of porphyrin-based coatings in AR14 water solution accelerates its degradation under light exposure, as its full oxidation occurs after 5h with the presence of **P3** (or **P4**)-based coatings, whereas more than 10 h is needed without coatings. The reusability of the **P4**-based materials for the photo-oxidation of AR14 has also been demonstrated, as the efficiency of the materials remains the same even after three cycles of irradiation.

## Figures and Tables

**Table 1:** Chemical structures of the photoinitiating systems, monomer, photoinitiating systems, and acid red 14 used in this study.

Name	Chemical Structure
**P3** 5,15-diphenyl-10,20-*bis*(4-bromophenyl) porphyrin	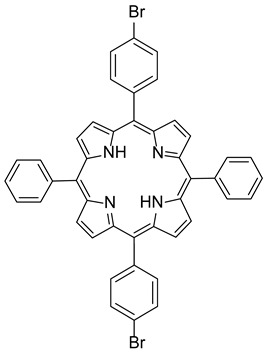
**P4** 5,15-diphenyl-10,20-*bis*(4-vinylphenyl) porphyrin	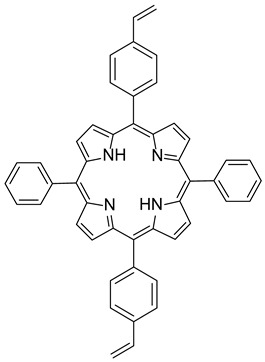
MDEA *N*-methyldiethanol amine	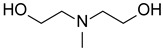
Iod *Bis*(4-methylphenyl) iodonium hexafluorophosphate	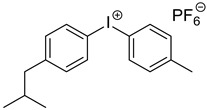
Cysteamine	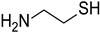
*N*-acetylcysteine (NAC)	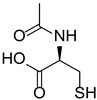
SOA Soybean oil acrylate	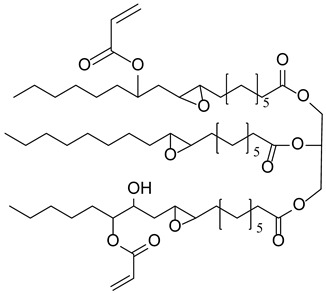
Acid red 14 Disodium 4-hydroxy-2-[(*E*)-(4-sulfonato-1-naphthyl)diazenyl]naphthalene-1-sulfonate	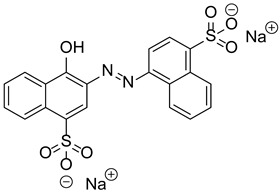

**Table 2:** Absorbance, emission, and phosphorescence properties of **P3** and **P4**.

Porphyrin	Soret Band: λ_abs/_nm (ε/M^−1^·cm^−1^)	Q Bands: λ_abs/_nm (ε/M^−1^·cm^−1^)	λ_em_/nm (Intensity/Arb·Unit)	λ_phos_/nm
**P3**	418 (62,825)	516 (4660)	653 (1), 717 (0.63)	867
		554 (2660)		
		588 (1993)		
		649 (1241)		
**P4**	419 (44,867)	515 (1867)	646 (1), 708 (0.86)	867
		551 (983)		
		589 (568)		
		646 (472)		

**Table 3:** Free-energy changes (in eV) at the singlet excited (ΔG_S_) and triplet excited state (ΔG_T_) for different photoinitiating systems involving **P3** and **P4** in the presence of MDEA, Iod, cysteamine, and *N*-acetylcysteine as co-initiators. ΔG_S_ and ΔG_T_ are in eV.

	MDEA		Iod		Cysteamine		*N*-Acetylcysteine (NAC)	
	ΔG_S_	ΔG_T_	ΔG_S_	ΔG_T_	ΔG_S_	ΔG_T_	ΔG_S_	ΔG_T_
**P3**	+0.04	+0.51	−0.11	+0.28	+0.24	+0.71	+0.11	+0.58
**P4**	−0.25	+0.23	−0.34	+0.07	−0.05	+0.43	−0.18	+0.30

**Table 4:** Final acrylate conversion (%) of SOA determined by IR after 800 s of irradiation under LEDs@385 nm, 405 nm, 455 nm, and 530 nm with different photoinitiating systems under laminate (a) and under air (b).

	Final Acrylate Conversion (%) of SOA							
Photoinitiating systems	385 nm		405 nm		455 nm		530 nm	
**P3**/MDEA	73^a^	55 ^b^	91 ^a^	93 ^b^	64 ^a^	40 ^b^	43 ^a^	26 ^b^
**P4**/MDEA	76 ^a^	48 ^b^	91 ^a^	80 ^b^	66 ^a^	40 ^b^	47 ^a^	26 ^b^
CQ/MDEA	28 ^a^	np ^b^	56 ^a^	38 ^b^	68 ^a^	44 ^b^	40 ^a^	np ^b^
**P3**/Iod	77 ^a^	30 ^b^	86 ^a^	46 ^b^	66 ^a^	13 ^b^	70 ^a^	13 ^b^
**P4**/Iod	62 ^a^	28 ^b^	70 ^a^	46 ^b^	51 ^a^	9 ^b^	43 ^a^	2 ^b^
CQ/Iod	79 ^a^	13 ^b^	84 ^a^	51 ^b^	81 ^a^	64 ^b^	73 ^a^	np ^b^
**P3**/cysteamine	47 ^a^	30 ^b^	61 ^a^	58 ^b^	29 ^a^	26 ^b^	21 ^a^	15
**P4**/cysteamine	45 ^a^	25 ^b^	70 ^a^	53 ^b^	31 ^a^	10 ^b^	17 ^a^	np ^b^
CQ/cysteamine	Polymerization at Rt							
**P3**/NAC	99 ^a^	37 ^b^	78 ^a^	96 ^b^	82 ^a^	np ^b^	50 ^a^	np ^b^
**P4**/NAC	92 ^a^	np ^b^	78 ^a^	85 ^b^	92 ^a^	np ^b^	61 ^a^	np ^b^
CQ/NAC	78 ^a^	14 ^b^	98 ^a^	33 ^b^	95 ^a^	44 ^b^	52 ^a^	np ^b^

^a^ under laminate, ^b^ under air, np: no polymerization.

## Data Availability

The original contributions presented in this study are included in the article/Supplementary Materials. Further inquiries can be directed to the corresponding author.

## Supplementary Materials

The following supporting information can be downloaded at: https://www.mdpi.com/article/10.3390/polym17212882/s1. Figure S1: ^1^H NMR spectrum of **2** in CDCl_3_. Figure S2: ^1^H NMR spectrum of **P3** in CDCl_3_. Figure S3: COSY ^1^H-^1^H NMR spectrum of **P3** in CDCl_3_. Figure S4: ^13^C NMR spectrum of **P3** in CDCl_3_. Figure S5: ^1^H NMR spectrum of **P4** in CDCl_3_. Figure S6: ^13^C NMR spectrum of **P4** in CDCl_3_. Figure S7: Normalized phosphorescence spectra of **P3** and **P4** recorded in a glassy matrix of 2-MTHF (λ_ex_. = 515 nm, delay = 1 ms, and time-gate = 40 ms). Insets: Time decays of the phosphorescence signals with their corresponding fitted curves. Figure S8: Cyclic voltammetry of 1) **P3** in DMF and 2) **P4** in DMF ([**P3**] = [**P4**] = [N_4_Et_4_BF_4_] = 10^−3^ M) between (A) 0 V and 2 V to determine the oxidation peak potential E_ox_ and (B) 0 V and −2 V to determine reduction peak potential E_red_. Figure S9: Steady-state photolysis of different photoinitiating systems under air after LED @405 nm exposure of (A) **P3** and (B) **P4**. [**P3**] = 1.85 × 10^−5^ M, [**P4**] = 2.9 × 10^−6^ M in DCM. Figure S10: The normalized experimental (1) and simulated (2) EPR spectrum obtained upon continuous in situ LED@400 nm exposure of (A) **P3** and (B) **P4** in chloroform under argon in the presence of DMPO spin trapping agent. Figure S11: Transient absorption spectra of (A) **P3** and (B) **P4** in a deoxygenated DCM solution after laser pulse (λ = 385 nm). Decay traces of (C) **P3** at 444 nm by LFP with and without O_2_ (τ = 25.7 μs without O_2_ and τ = 0.95 μs with O_2_) and (D) **P4** at 460 nm by LFP with and without O_2_ (τ = 28.2 μs without O_2_ and τ = 0.63 μs with O_2_). [**P3**] = 7.0 × 10^−5^ M; [**P4**] = 3.0 × 10^−5^ M. Figure S12: Quenching fluorescence of (A) **P3** and (B) **P4** after a gradual addition of MDEA. [**P3**] = 4.63 × 10^−6^ mol/L, [**P4**] = 6.17 × 10^−7^ M. Insert: Corresponding Stern–Volmer plot (K_SV_**^P3^**^/MDEA^ = 9.8 M^−1^, K_SV_**^P4^**^/MDEA^ = 0.9 M^−1^). Excitation at λ = 649 and 646 nm for **P3** and **P4**, respectively. Figure S13: Decay traces of (A) **P3** and (B) **P4** triplet state after a laser pulse (λ_ex_ = 385 nm) with a gradual addition of MDEA. [**P3**] = 7.0 × 10^−5^ M; [**P4**] = 3.0 × 10^−5^ M, [MDEA] = 1.5 × 10^−1^ M in DCM. Figure S14: The normalized experimental (1) and simulated (2) EPR spectra obtained upon continuous in situ LED@400 nm exposure of porphyrin derivatives in chloroform under argon in the presence of DMPO spin trapping agent and MDEA: (A) **P3** and (B) **P4**. Figure S15: Quenching fluorescence of (A) **P3** and (B) **P4** after a gradual addition of Iod. [**P3**] = 4.63 × 10^−6^ M, [**P4**] = 6.17 × 10^−7^ M. Corresponding Stern–Volmer plot for (C) **P3** (K_SV_**^P3^**^/IOD^ = 2600 M^−1^) and (D) **P4** (K_SV_**^P4^**^/IOD^ = 1756 M^−1^). Excitation at λ = 649 and 646 nm for **P3** and **P4**, respectively. Figure S16: Decay traces of (A) **P3** and (B) **P4** triplet state after a laser pulse (λ_ex_ = 385 nm) with a gradual addition of Iod. [**P3**] = 7.0 × 10^−5^ M; [**P4**] = 3.0 × 10^−5^ M, [Iod] = 2.2 × 10^−3^ M in DCM. Figure S17: The normalized experimental (1) and simulated (2) EPR spectra obtained upon continuous in situ LED@400 nm exposure of porphyrin derivatives in chloroform under argon in the presence Iod: (A) **P3** and (B) **P4**. Figure S18: Steady-state photolysis of (A) **P3** and (B) **P4** in presence of cysteamine under air after LED @405 nm exposure. [**P3**] = 1.85 × 10^−5^ M, [**P4**] = 2.9 × 10^−6^ M, [cysteamine] = 1.7 × 10^−4^ M. Solvent = DCM. Figure S19: Quenching fluorescence of (A) **P3** and (B) **P4** after a gradual addition of cysteamine. [**P3**] = 4.63 × 10^−6^ M, [**P4**] = 6.17 × 10^−7^ M. Corresponding Stern–Volmer plot for (C) **P3** (K_SV_**^P3^**^/cysteamine^ = 235 M^−1^) and (D) **P4** (K_SV_**^P4^**^/cysteamine^ = 351 M^−1^). Excitation at λ = 649 and 646 nm for **P3** and **P4**, respectively. Figure S20: The normalized experimental (1) and simulated (2) EPR spectra obtained upon continuous in situ LED@400 nm exposure of porphyrin derivatives in chloroform under argon in the presence of DMPO spin trapping agent and cysteamine: (A) **P3** and (B) **P4**. Figure S21: Decay traces of (A) **P3** and (B) **P4** triplet state after a laser pulse (λ_ex_ = 385 nm) with a gradual addition of cysteamine. [**P3**] = 7.0 × 10^−5^ M; [**P4**] = 3.0 × 10^−5^ M, [cysteamine] = 1.3 × 10^−1^ M in DCM. Figure S22: Steady-state photolysis of (A) **P3** and (B) **P4** in presence of NAC under air after LED@405 nm exposure. [**P3**] = 1.85 × 10^−5^ M, [**P4**] = 2.9 × 10^−6^ M, [NAC] = 1.1 × 10^−4^ M. Solvent = DCM. Figure S23: Quenching fluorescence of (A) **P3** and (B) **P4** after a gradual addition of NAC. [**P3**] = 4.63 × 10^−6^ M, [**P4**] = 6.17 × 10^−7^ M. Corresponding Stern–Volmer plot for (C) **P3** (K_SV_**^P3^**^/NAC^ = 216 M^−1^). Excitation at λ = 649 and 646 nm for **P3** and **P4**, respectively. Figure S24: Decay traces of (A) **P3** and (B) **P4** triplet state after a laser pulse (λ_ex_ = 385 nm) with a gradual addition of NAC. [**P3**] = 7.0 × 10^−5^ M; [**P4**] = 3.0 × 10^−5^ M, [NAC] = 8.3 × 10^−2^ M in DCM. Figure S25: The normalized experimental (1) and simulated (2) EPR spectra obtained upon continuous in situ LED@400 nm exposure of porphyrin derivatives in chloroform under argon in the presence of DMPO spin trapping agent and NAC: (A) **P3** and (B) **P4**. Figure S26: Forrester–Hepburn mechanism. Figure S27: Oxidation of TPCPD in the presence of singlet oxygen. Figure S28: Adsorption of AR14 on the surface of **P3** (or **P4)**-based coating without light. The controls without coating and without light are presented to control the stability of the solution. Figure S29: UV–visible spectra of **P4**-based coating (a) before and (b) after three cycling processes of AR14 photodegradation under UV irradiation (UV lamp, λ_max_ = 365 nm). Figure S30: Photolysis of a DCM solution of **P4** under LED@405 nm irradiation; Table S1: Excitation energies (λ) and oscillator strengths (f) for different excited state transitions of **P3** and **P4** determined by TD-DFT using B3LYP/6-31G(d) basis set; Table S2: Photochemical properties of porphyrins. ^a^determined by peak potential using cyclic voltammetry. ^b^ calculated from the wavelength at the intersection of the absorption and emission spectra (λ_inter_), according to E_S_ = 1240/λ_inter_. ^c^ from the phosphorescence experiments; Table S3: Optimized ground state coordinates of **P3** obtained at the B3LYP/6-31G(d) level of theory; Table S4: Optimized ground state coordinates of **P4** obtained at the B3LYP/6-31G(d) level of theory; Table S5: Rate of polymerization of SOA determined by IR after the first seconds of irradiation under LEDs@385 nm, 405 nm, 455 nm and 530 nm with different photoinitiating systems under laminate (a) and under air (b); Equation S1: Determination of energy change at singlet ΔG_S_ or triplet state ΔG_T_ of the electron transfer between a donor and an acceptor under irradiation. Where F is the Faraday constant, E_ox_ is the oxidation potential of the donor, E_red_ is the reduction potential of the acceptor, E_S (or T)_ is the transition energy from the excited singlet (or triplet) state of the photoinitiator (**P3** or **P4**) to the ground state. Reference [86] are cited in the supplementary materials.

## Abbreviations

The following abbreviations are used in this manuscript:

AOP	Advanced oxidation process
AR14	Acid red 14
DCM	Dichloromethane
DDQ	2,3-dichloro-5,6-dicyano-1,4-benzoquinone
DFT	Density Functional Theory
DMPO	5,5-dimethylpyrroline-N-oxide
EPR ST	Electron paramagnetic resonance spin trapping
FRP	Free-radical photopolymerization
Iod	4-(2-methylpropyl)phenyliodonium hexafluorophosphate
LED	Light-emitting diode
MDEA	N-methyldiethanolamine
NAC	N-acetylcysteine
NBS	N-bromosuccinimide
NMR	Nuclear Magnetic Resonance
PET-RAFT	Photoinduced electron/energy transfer reversible addition–fragmentation chain transfer
POP	Porous organic polymer
ROS	Reactive oxygen species
RT-FTIR	Real-Time Fourier-Transformed Infrared spectroscopy
SOA	Soybean oil acrylate
TD-DFT	Time-Dependent Density Functional Theory
TFA	Trifluoroacetic acid
THF	Tetrahydrofuran
TLC	Thin-layer chromatography
TPCPD	Tetraphenylcyclopentadienone
UV	Ultraviolet
