# Supplementary material for: Porphyrin-Based Bio-Sourced Materials for Water Depollution Under Light Exposure
Source: Polymers (Basel). 2025 Oct 29;17(21):2882. doi: 10.3390/polym17212882 (PMC12608407; doi:10.3390/polym17212882)
Supplement: Supplementary file 1 [file polymers-17-02882-s001.zip › polymers-3877346-supplementary.pdf]

SUPPORTING INFORMATION

Porphyrin-based biosourced materials for water depollution under light exposure

Fanny Schnetz[1], Marc Presset[1], Jean-Pierre Malval[2], Yamin Leprince-Wang[3], Isabelle Navizet[4], Davy-Louis Versace[1]*

1University Paris-Est Creteil, CNRS, ICMPE, UMR 7182, 94320 Thiais, France

2Institut de Science des Matériaux de Mulhouse, UMR CNRS 7361, Université de Haute Alsace, 15 rue Jean Starcky, 68057 Mulhouse, France

[3]LGE, Université Gustave Eiffel, F-77454 Marne-la-Vallée, France.

[4]Univ Gustave Eiffel, Univ Paris Est Creteil, CNRS, UMR 8208, MSME, F-77454 Marne-la-Vallée, France.

FIGURES

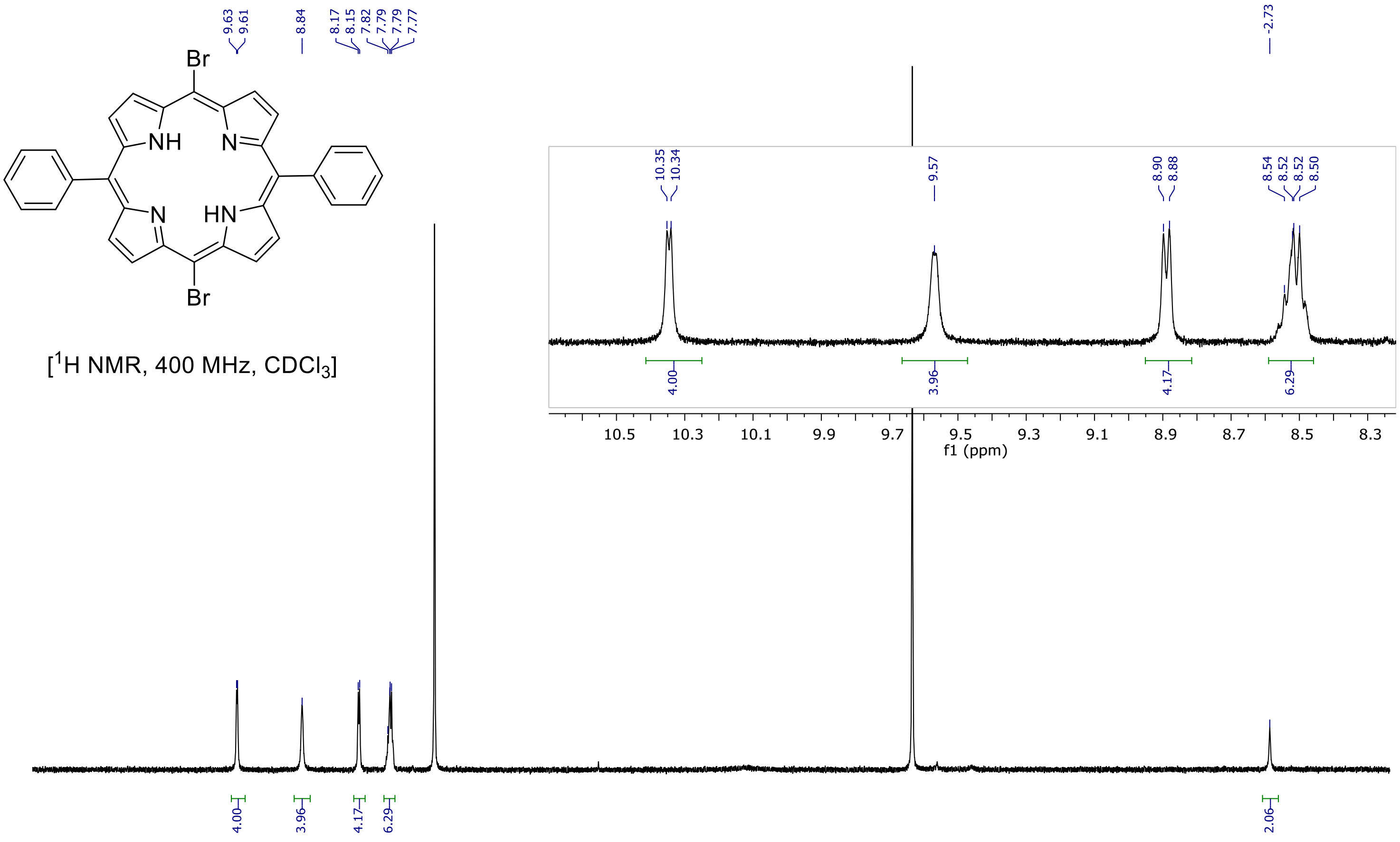

Figure S1: *^{1}H NMR spectrum of **2** in $CDCl_3$.*

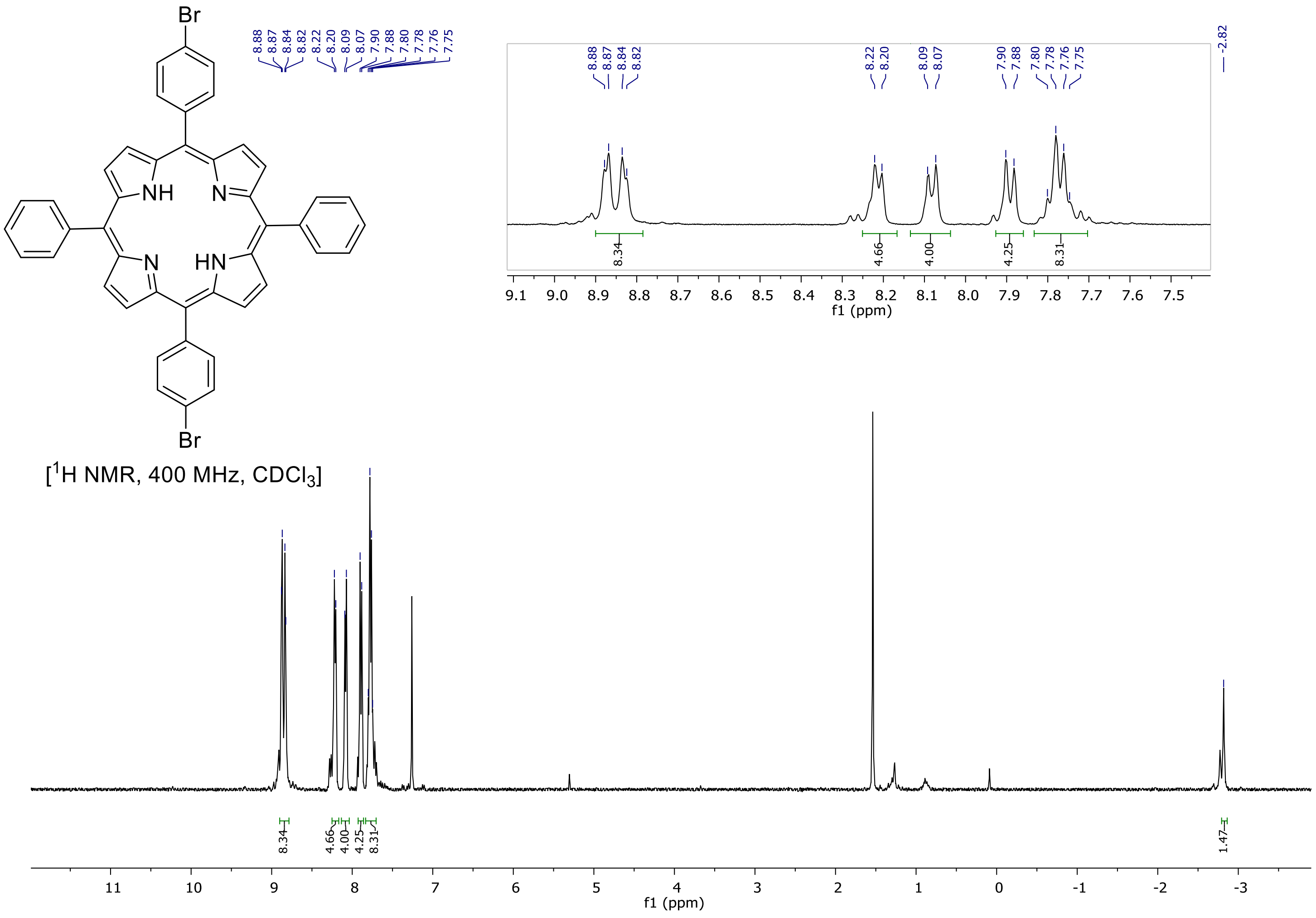

__Figure S2__: ^{1}H NMR spectrum of __P3__ in $CDCl_3$.

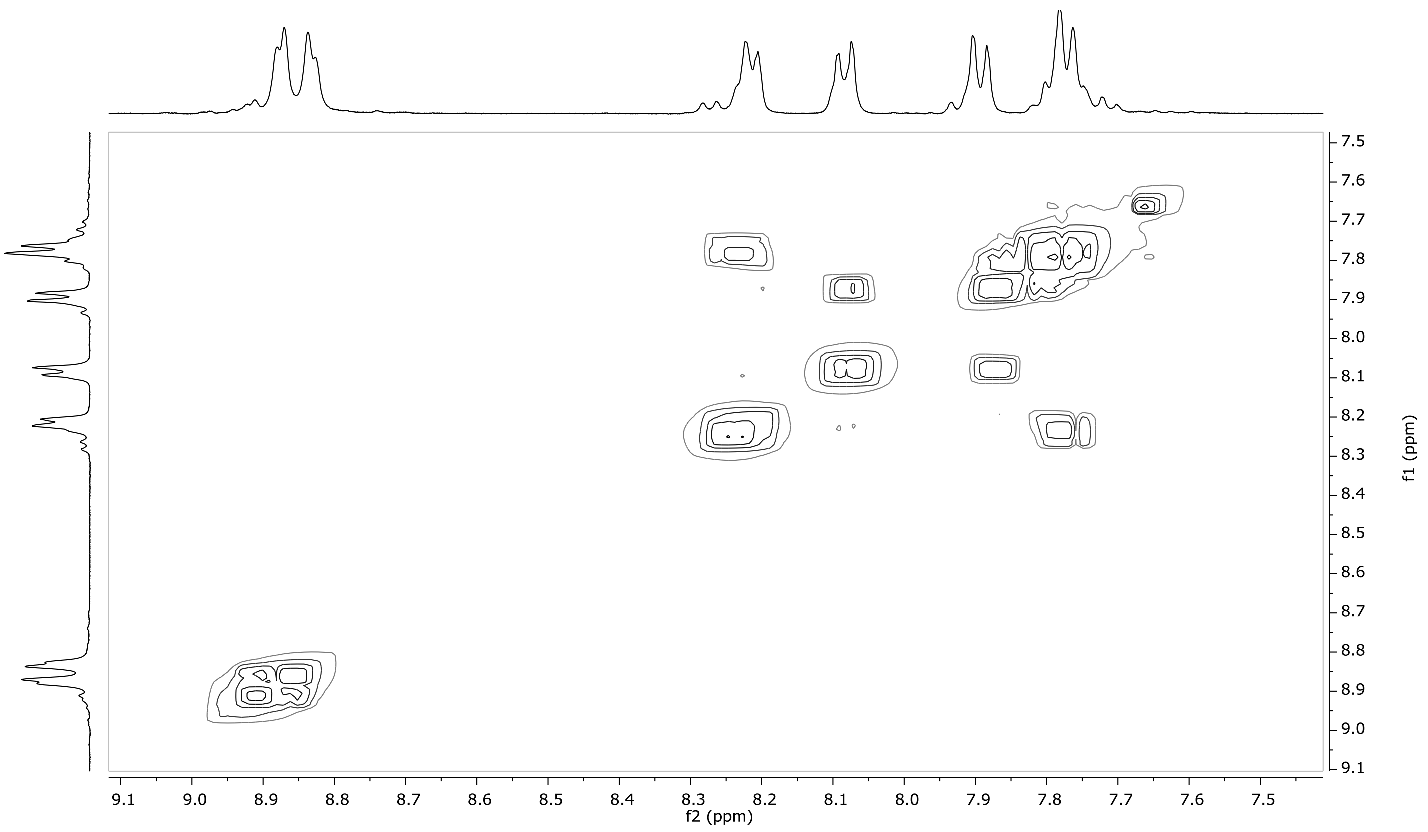

__Figure S3:__ COSY ^{1}H-^{1}H NMR spectrum of __P3__ in $CDCl_3$.

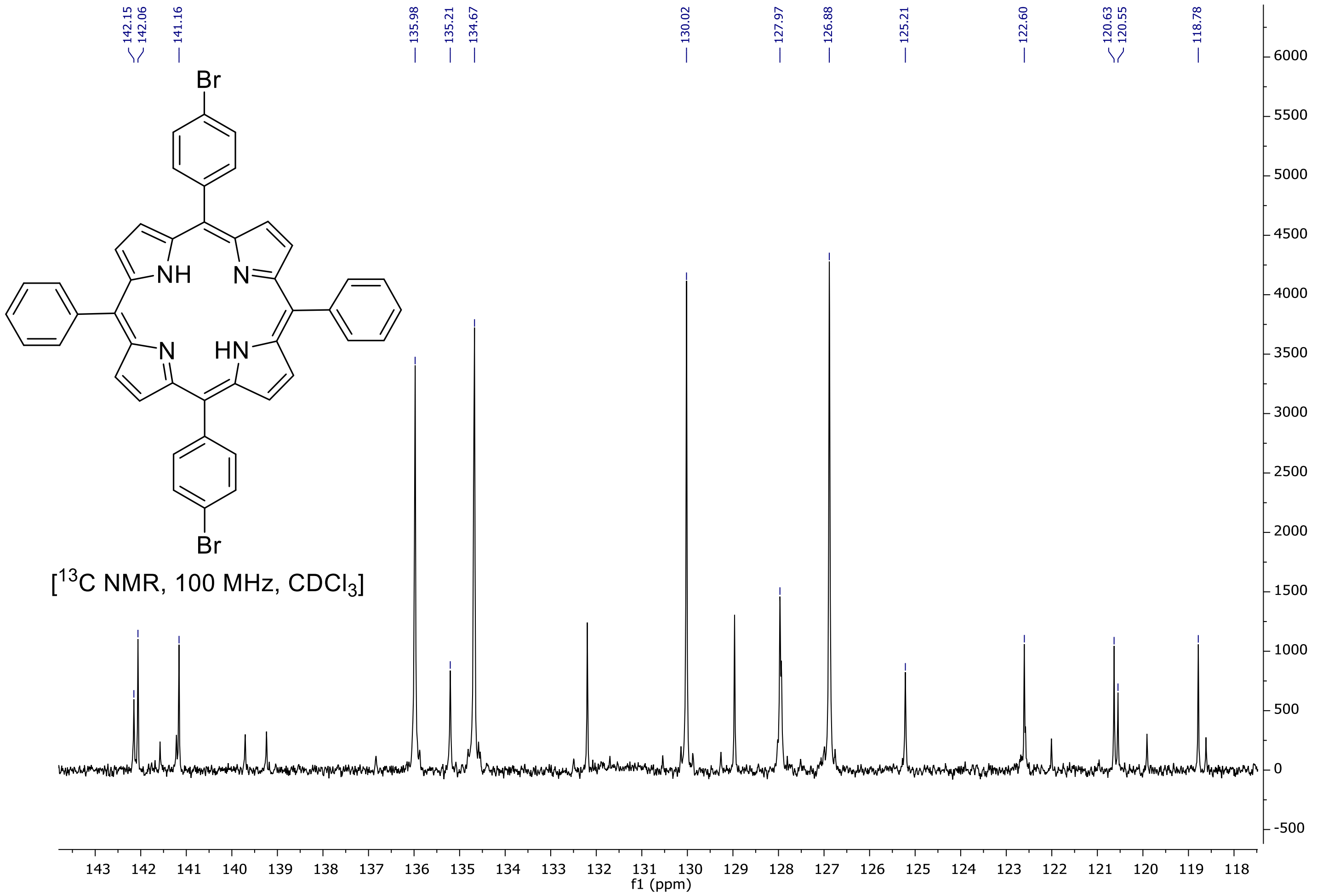

Figure S4: *^{13}C NMR spectrum of **P3** in $CDCl_3$.*

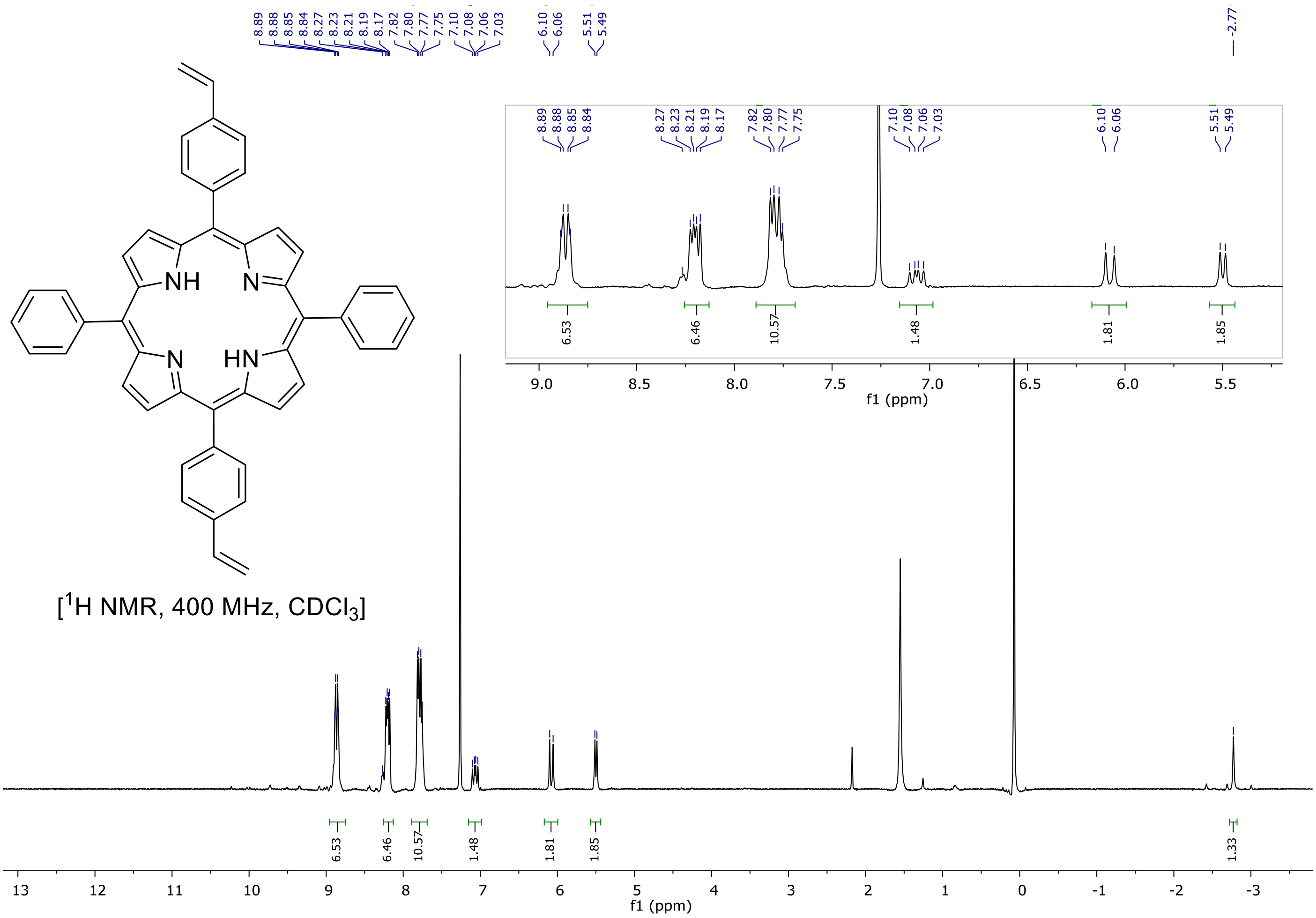

Figure S5: *^{1}H NMR spectrum of **P4** in $CDCl_3$.*

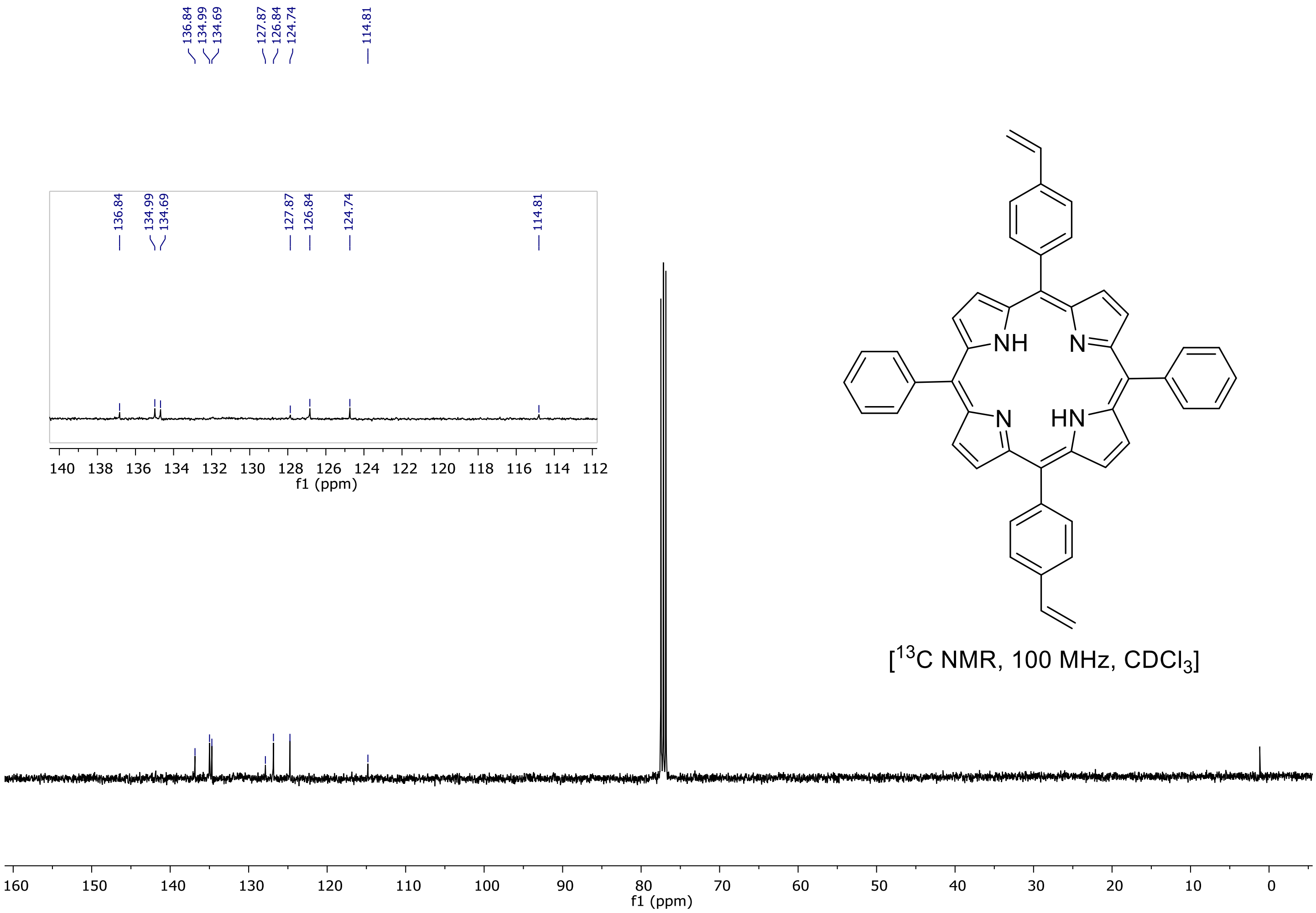

***Figure S6**: ^{13}C NMR spectrum of **P4** in $CDCl_3$.*

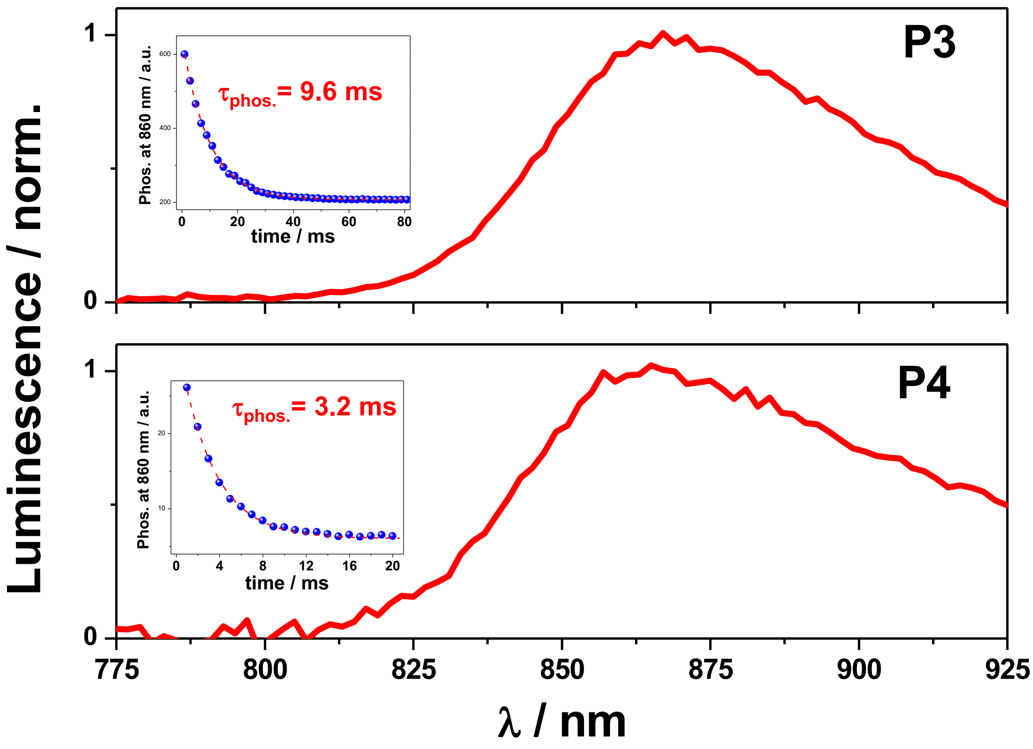

***Figure S7**: Normalized phosphorescence spectra of **P3** and **P4** recorded in a glassy matrix of 2-MTHF ($\lambda_{ex.}$ = 515 nm, delay = 1 ms, and time-gate = 40 ms). Insets: Time decays of the phosphorescence signals with their corresponding fitted curves.*

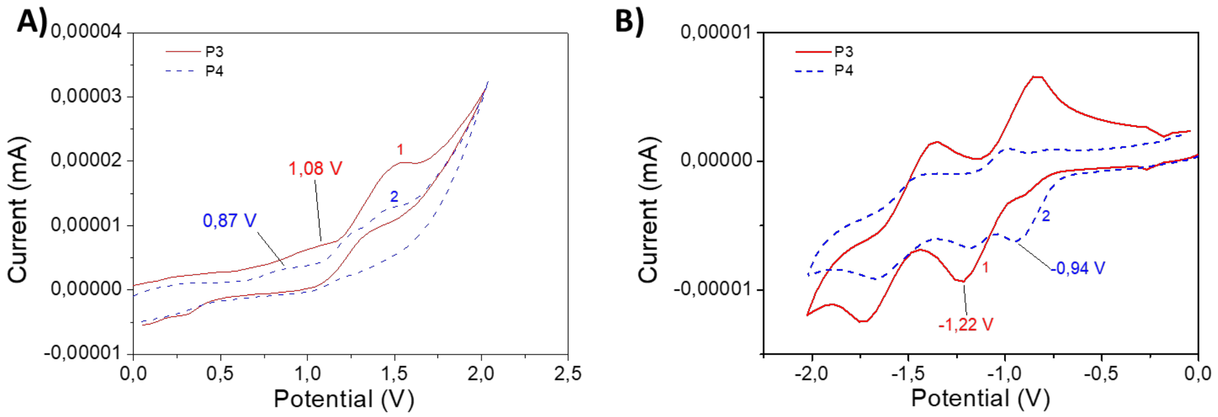

Figure S8*: Cyclic voltammetry of 1)* ***P3*** *in DMF and 2)* ***P4*** *in DMF ([****P3****] = [****P4****] = [$N_4Et_4BF_4$] = 10^{-3} M) between* ***A)*** *0 V and 2 V to determine the oxidation peak potential E_{ox} and* ***B)*** *0 V and -2 V to determine reduction peak potential E_{red}.*

Photostability of porphyrin.

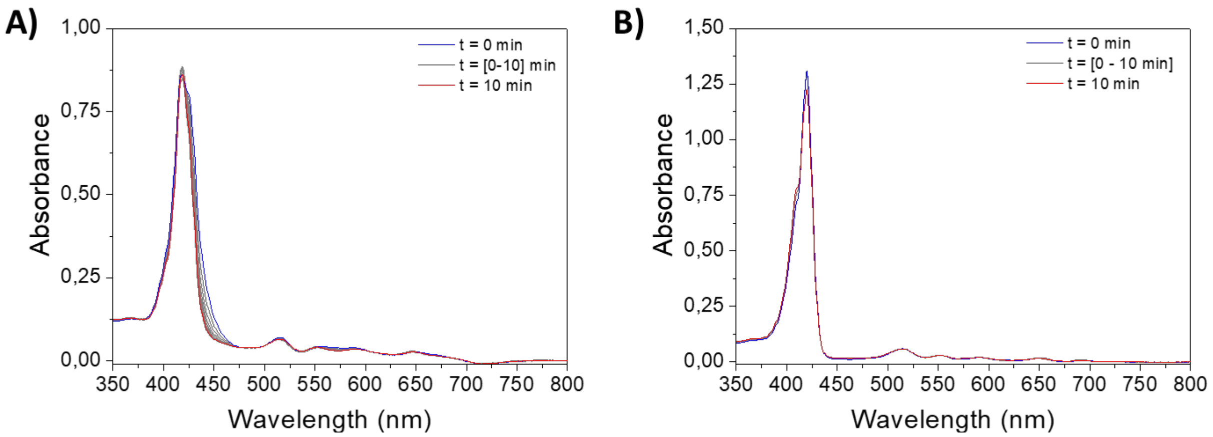

Figure S9*: Steady state photolysis of different photoinitiating systems under air after LED @405 nm exposure of* ***A) P3*** *and* ***B) P4****. [****P3****] = 1.85 x 10^{-5} M, [****P4****] = 2.9 x 10^{-6} M in DCM.*

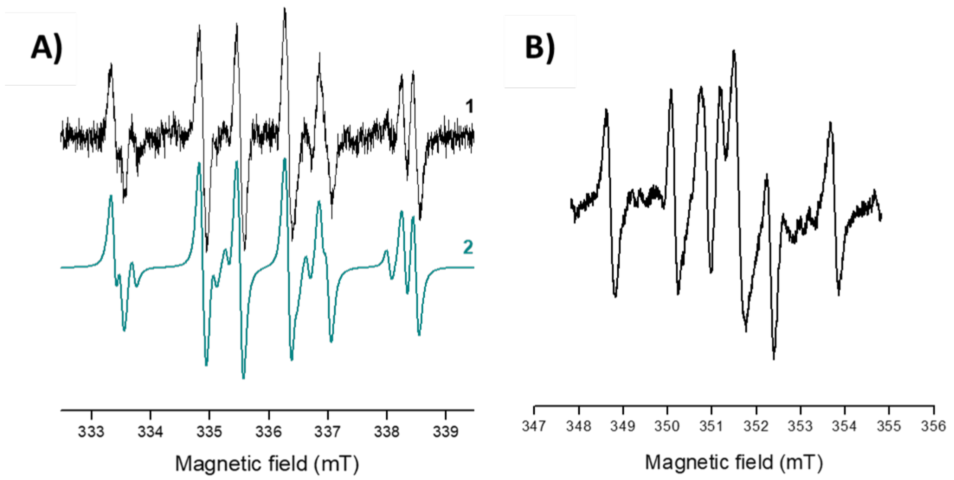

Figure S10*: The normalized experimental (1) and simulated (2) EPR spectrum obtained upon continuous in situ LED@400 nm exposure of* ***A) P3*** *and* ***B) P4*** *in chloroform under argon in the presence of DMPO spin trapping agent.*

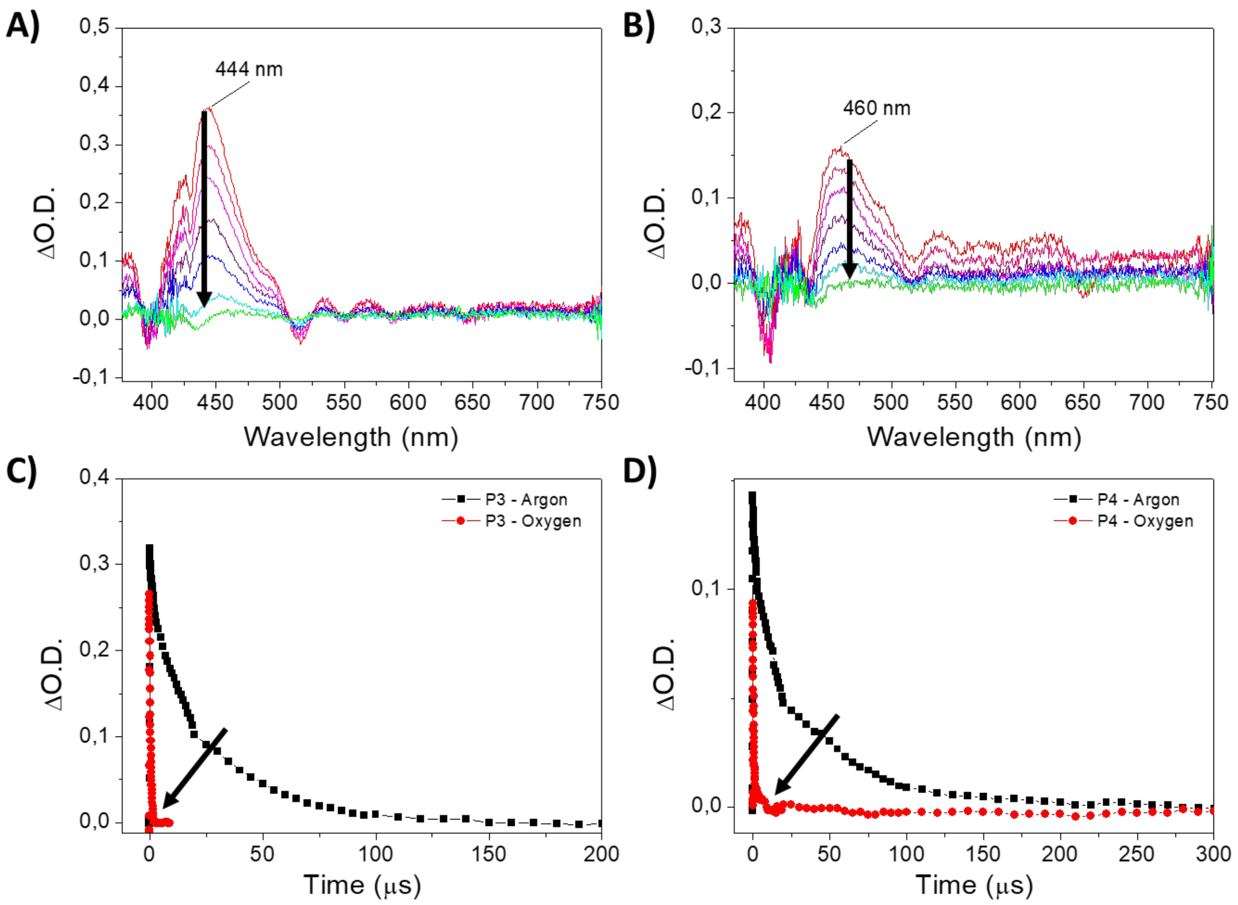

***Figure S11**: Transient absorption spectra of **A)** **P3** and **B)** **P4** in a deoxygenated DCM solution after laser pulse (λ = 385 nm). Decay traces of **C)** **P3** at 444 nm by LFP with and without O_2 (τ = 25.7 μs without O_2 and τ = 0.95 μs with O_2) and **D)** **P4** at 460 nm by LFP with and without O_2 (τ = 28.2 μs without O_2 and τ = 0.63 μs with O_2). [**P3**] = 7.0 x 10^{-5} M; [**P4**] = 3.0 x 10^{-5} M.*

Reactivity with MDEA:

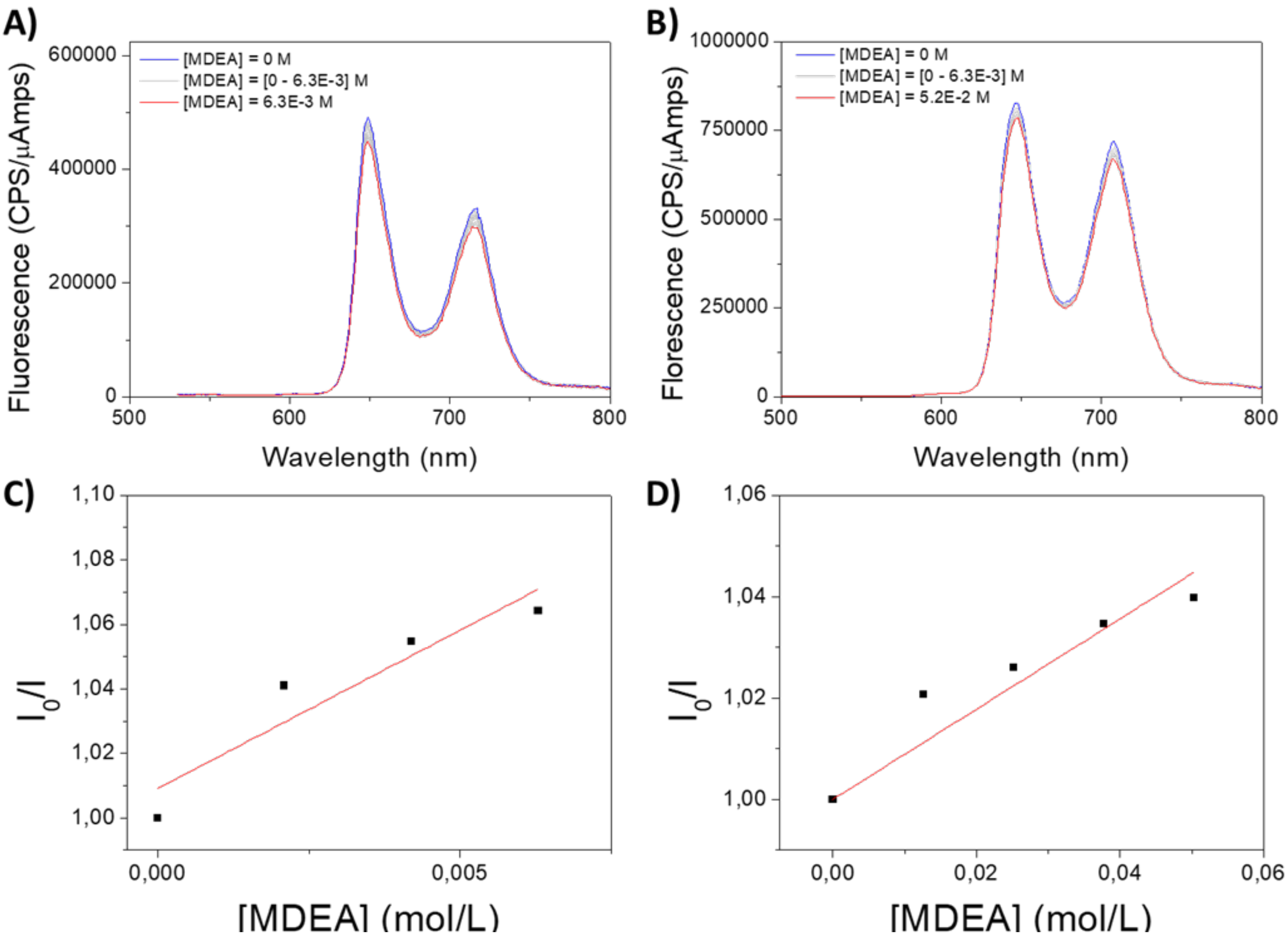

***Figure S12**: Quenching fluorescence of **A)** **P3** and **B)** **P4** after a gradual addition of MDEA. [**P3**] = 4.63 x 10-6 mol/L, [**P4**] = 6.17 x 10^{-7} M. Insert: Corresponding Stern-Volmer plot ($K_{SV}^{P3/MDEA}$ = 9.8 M^{-1}, $K_{SV}^{P4/MDEA}$ = 0.9 M^{-1}). Excitation at λ = 649 and 646 nm for **P3** and **P4** respectively.*

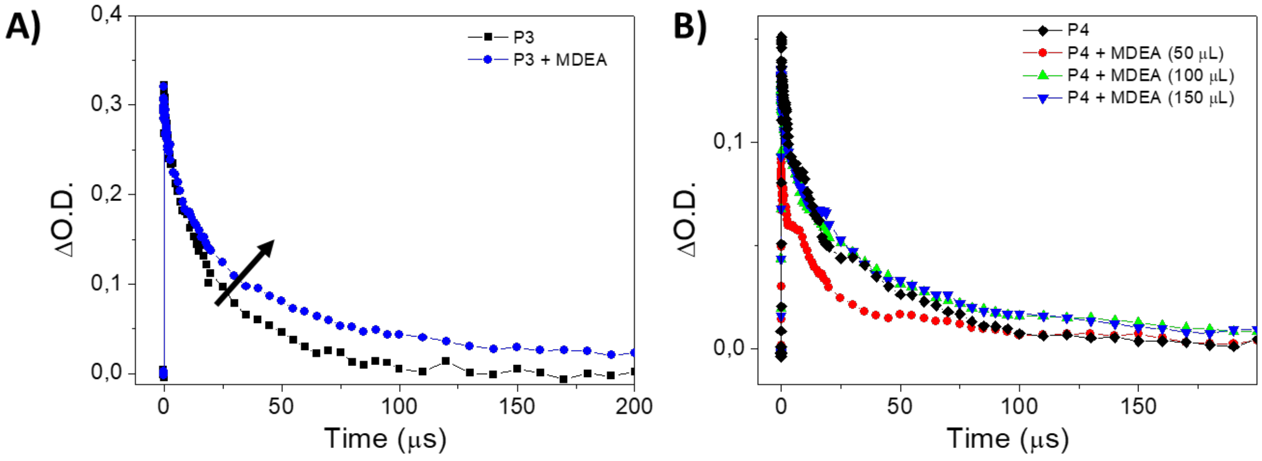

***Figure S13**: Decay traces of **A)** **P3** and **B)** **P4** triplet state after a laser pulse (λ_{ex} = 385 nm) with a gradual addition of MDEA. [**P3**] = 7.0 x 10^{-5} M; [**P4**] = 3.0 x 10^{-5} M, [MDEA] = 1.5 x 10^{-1} M in DCM.*

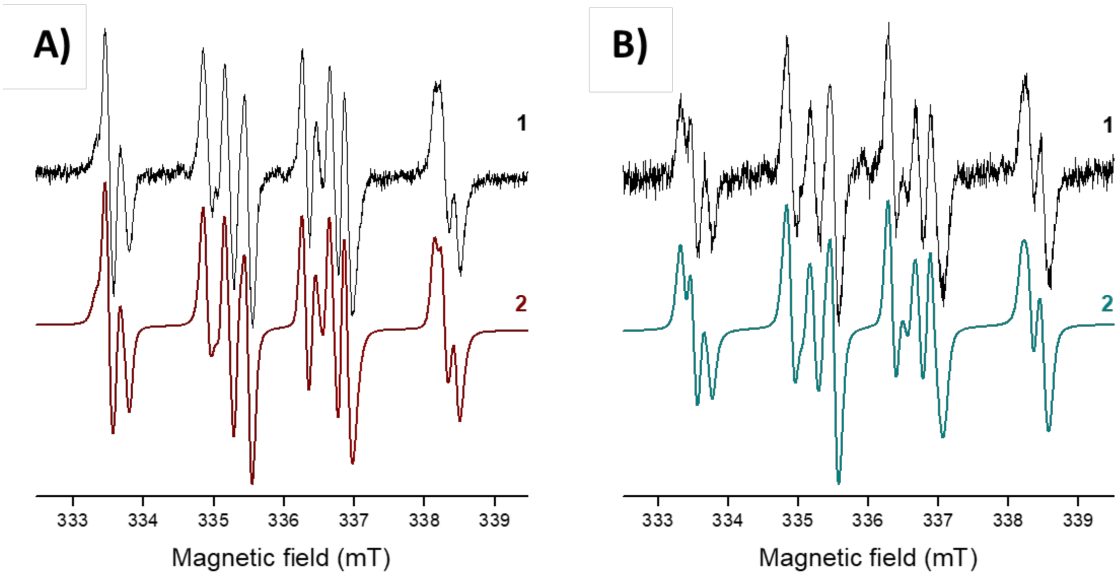

Figure S14: *The normalized experimental (1) and simulated (2) EPR spectra obtained upon continuous in situ LED@400 nm exposure of porphyrin derivatives in chloroform under argon in the presence of DMPO spin trapping agent and MDEA:* ***A)*** ***P3*** *and* ***B)*** ***P4****.*

Reactivity with Iod:

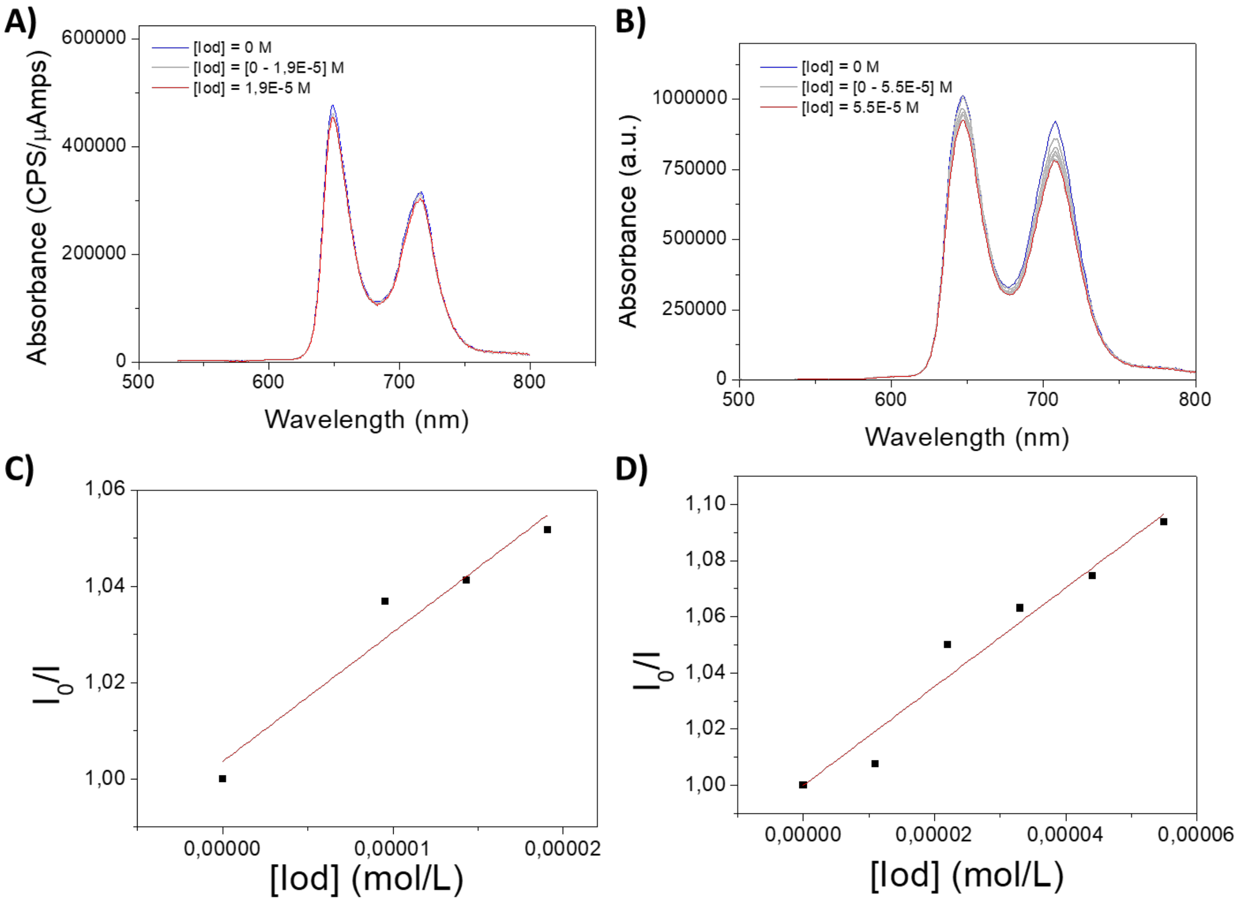

Figure S15*: Quenching fluorescence of* ***A)*** ***P3*** *and* ***B)*** ***P4*** *after a gradual addition of Iod. [****P3****] = 4.63 x 10^{-6} M, [****P4****] = 6.17 x 10^{-7} M. Corresponding Stern-Volmer plot for* ***C)*** ***P3*** *($K_{SV}^{P3/IOD}$ = 2600 M^{-1}) and* ***D)*** ***P4*** *($K_{SV}^{P4/IOD}$ = 1756 M^{-1}). Excitation at λ = 649 and 646 nm for* ***P3*** *and* ***P4*** *respectively.*

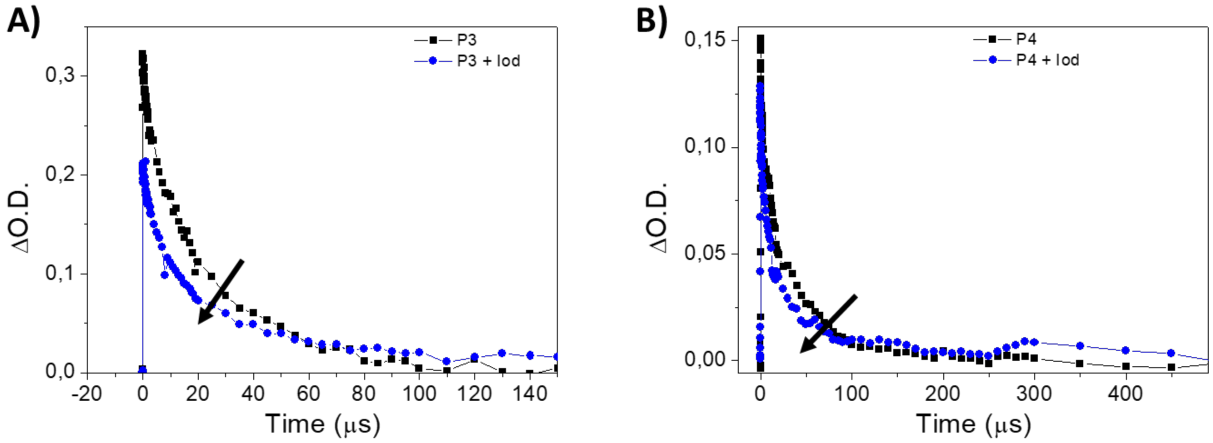

Figure S16: *Decay traces of* ***A)*** ***P3*** *and* ***B)*** ***P4*** *triplet state after a laser pulse (λ_{ex} = 385 nm) with a gradual addition of Iod. [****P3****] = 7.0 x 10^{-5} M; [****P4****] = 3.0 x 10^{-5} M, [Iod] = 2.2 x 10^{-3} M in DCM.*

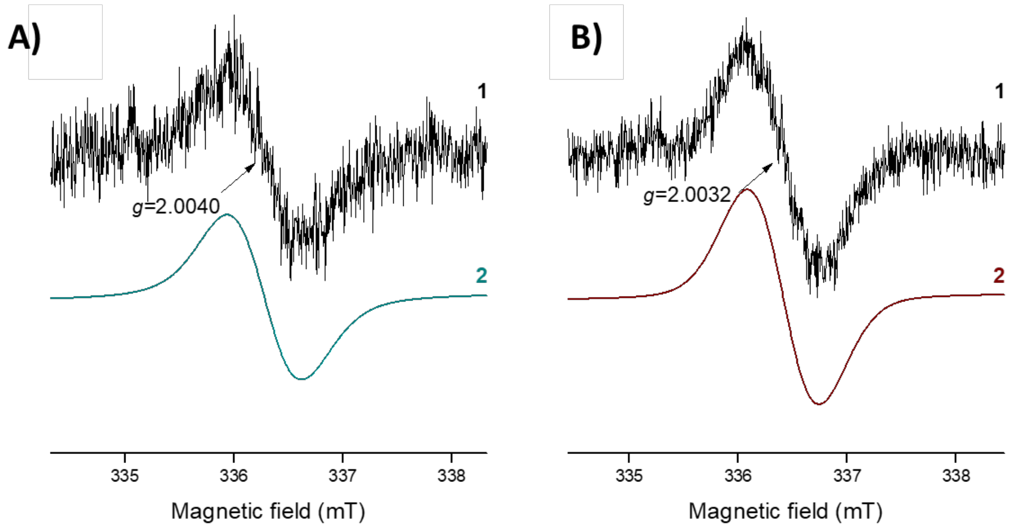

Figure S17: *The normalized experimental (1) and simulated (2) EPR spectra obtained upon continuous in situ LED@400 nm exposure of porphyrin derivatives in chloroform under argon in the presence Iod:* ***A)*** ***P3*** *and* ***B)*** ***P4****.*

Reactivity with cysteamine:

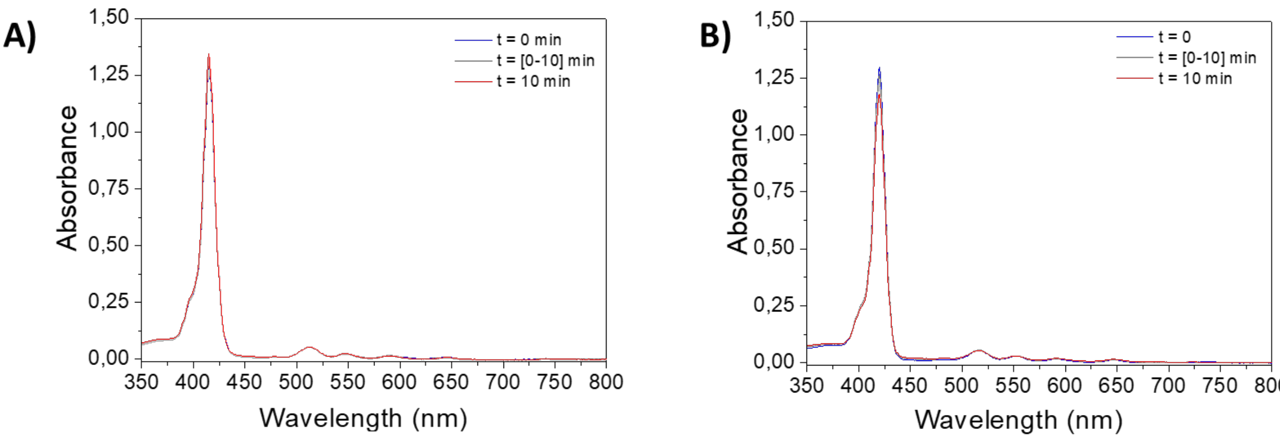

Figure S18: *Steady state photolysis of* ***A)*** ***P3*** *and* ***B)*** ***P4*** *in presence of cysteamine under air after LED @405 nm exposure. [****P3****] = 1.85 x 10^{-5} M, [****P4****] = 2.9 x 10^{-6} M, [cysteamine] = 1.7 x 10^{-4} M. Solvent = DCM*

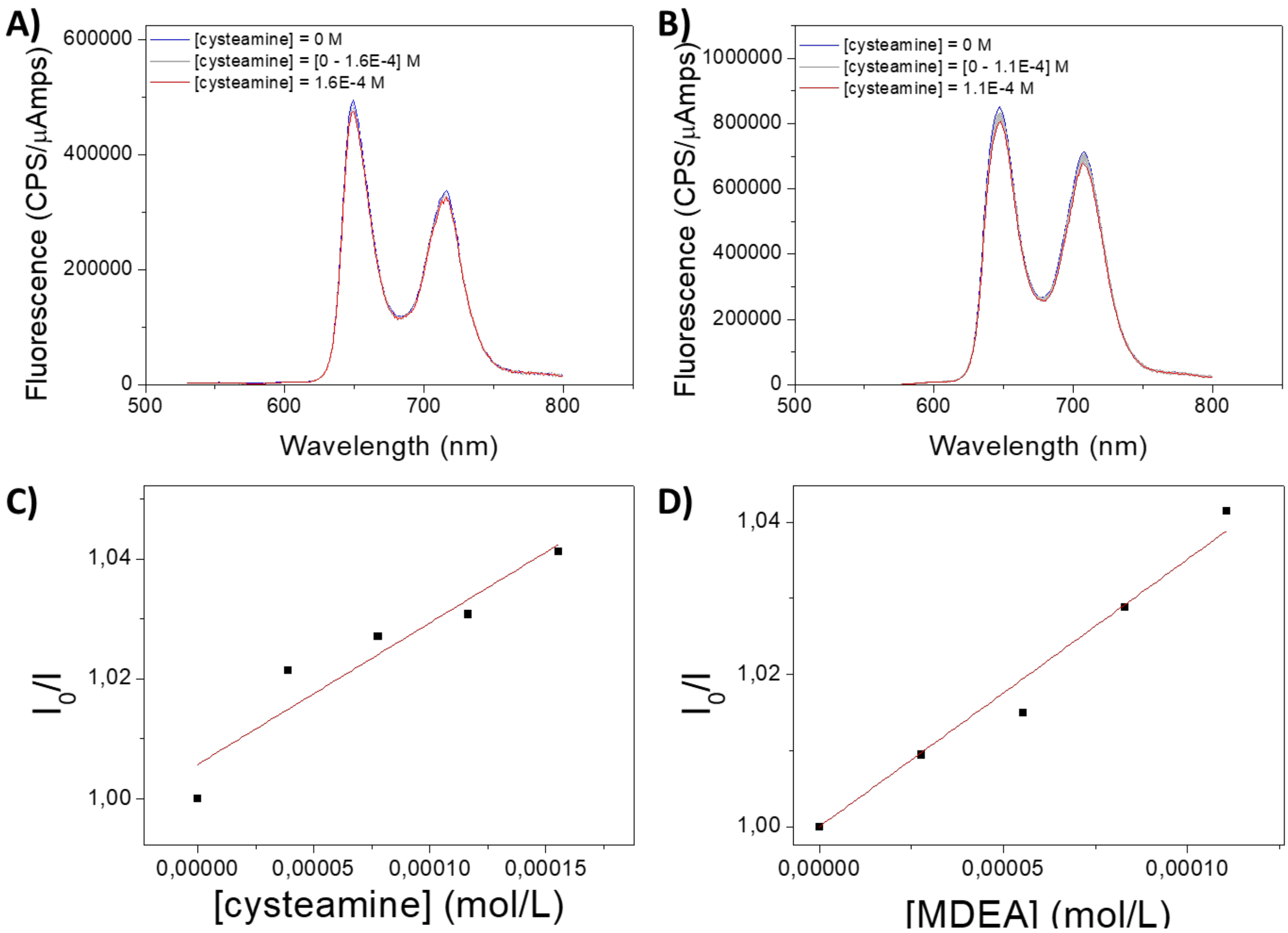

***Figure S19**: Quenching fluorescence of **A)** **P3** and **B)** **P4** after a gradual addition of cysteamine. [**P3**] = 4.63 x 10^{-6} M, [**P4**] = 6.17 x 10^{-7} M. Corresponding Stern-Volmer plot for **C)** **P3** ($K_{SV}^{P3/cysteamine}$ = 235 M^{-1}) and **D)** **P4** ($K_{SV}^{P4/cysteamine}$ = 351 M^{-1}). Excitation at λ = 649 and 646 nm for **P3** and **P4** respectively.*

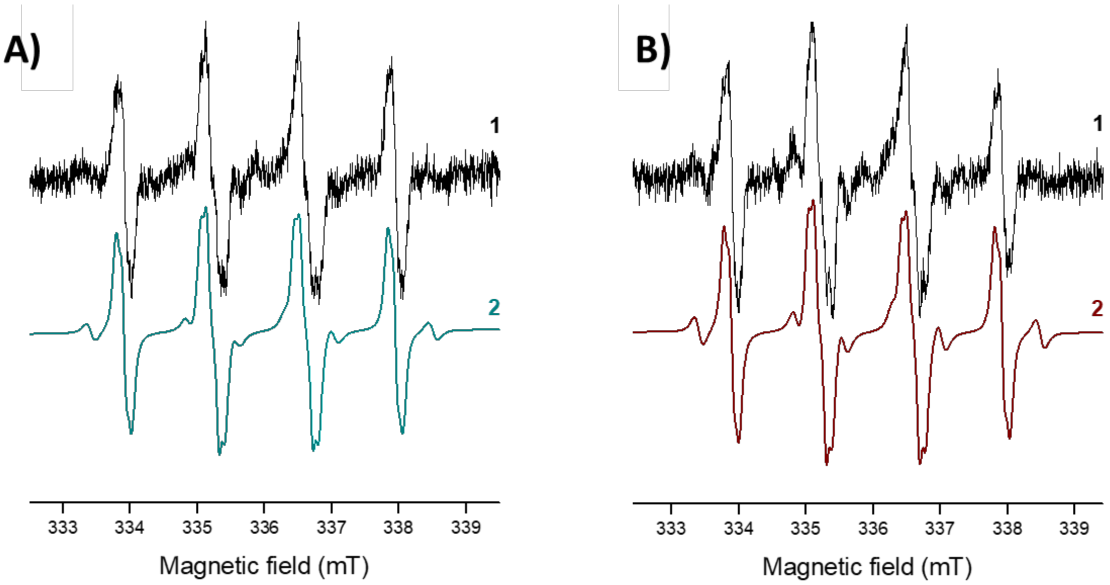

***Figure S20:** The normalized experimental (1) and simulated (2) EPR spectra obtained upon continuous in situ LED@400 nm exposure of porphyrin derivatives in chloroform under argon in the presence of DMPO spin trapping agent and cysteamine: **A)** **P3** and **B)** **P4**.*

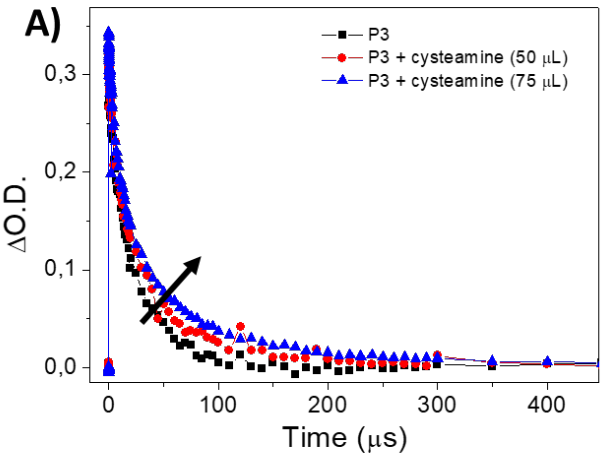

***Figure S21**: Decay traces of **A**) **P3** and **B**) **P4** triplet state after a laser pulse (λ_{ex} = 385 nm) with a gradual addition of cysteamine. [**P3**] = 7.0 x 10^{-5} M; [**P4**] = 3.0 x 10^{-5} M, [cysteamine] = 1.3 x 10^{-1} M in DCM.*

Reactivity with *N*-acetylcysteine (NAC):

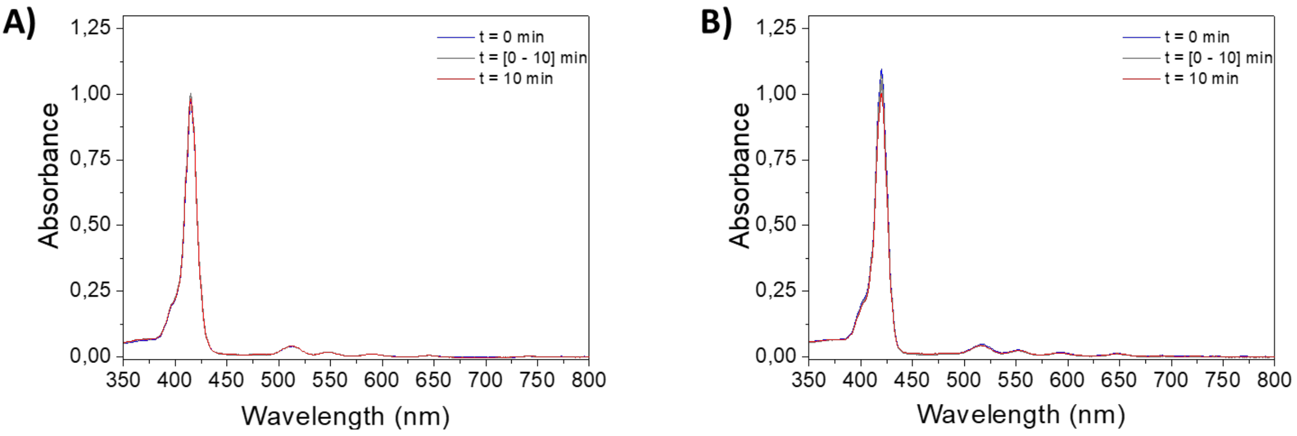

***Figure S22**: Steady state photolysis of **A**) **P3** and **B**) **P4** in presence of NAC under air after LED @405 nm exposure. [**P3**] = 1.85 x 10^{-5} M, [**P4**] = 2.9 x 10^{-6} M, [NAC] = 1.1 x 10^{-4} M. Solvent = DCM*

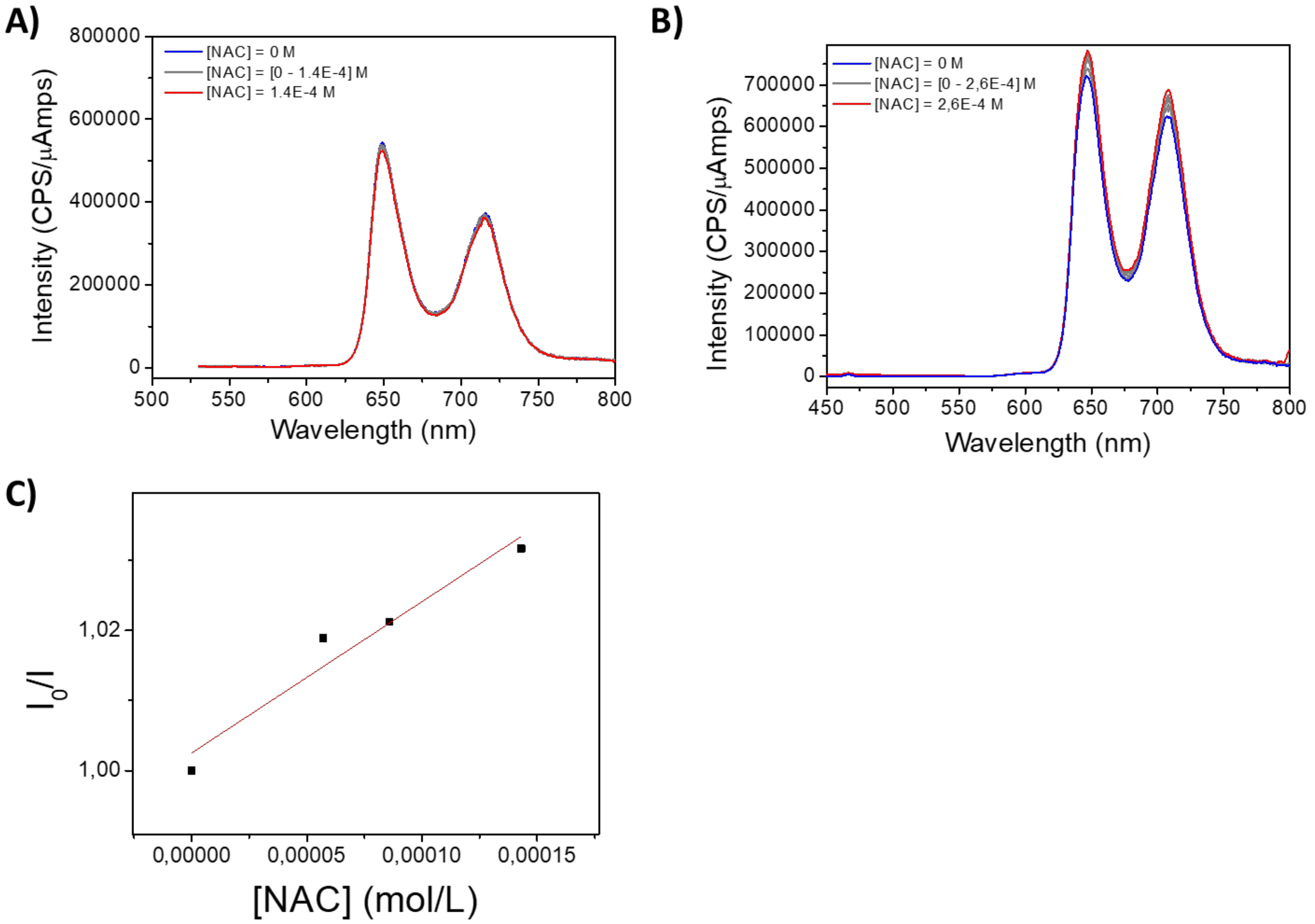

***Figure S23**: Quenching fluorescence of **A)** **P3** and **B)** **P4** after a gradual addition of NAC. [**P3**] = 4.63 x 10^{-6} M, [**P4**] = 6.17 x 10^{-7} M. Corresponding Stern-Volmer plot for **C)** **P3** ($K_{SV}^{P3/NAC}$ = 216 M^{-1}). Excitation at λ = 649 and 646 nm for **P3** and **P4** respectively.*

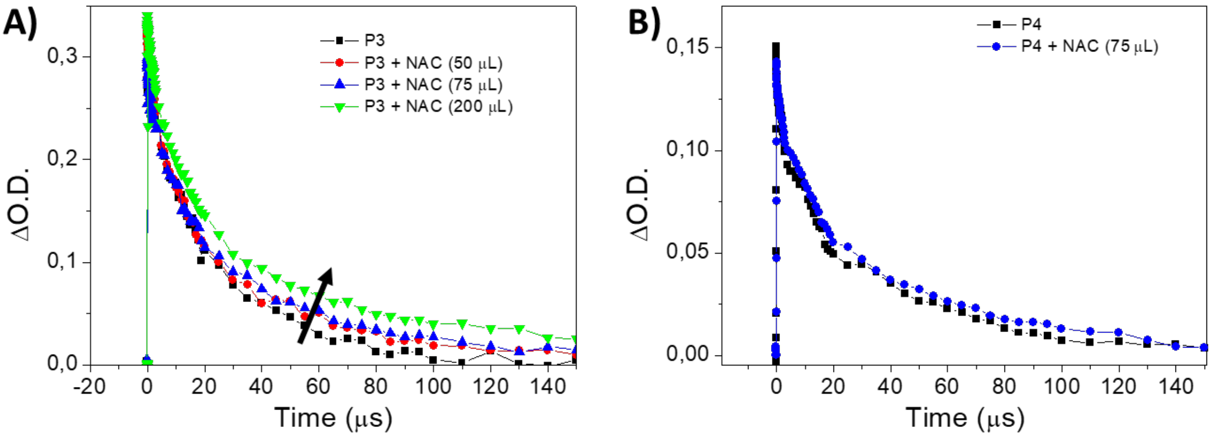

***Figure S24**: Decay traces of **A)** **P3** and **B)** **P4** triplet state after a laser pulse (λ_{ex} = 385 nm) with a gradual addition of NAC. [**P3**] = 7.0 x 10^{-5} M; [**P4**] = 3.0 x 10^{-5} M, [NAC] = 8.3 x 10^{-2} M in DCM.*

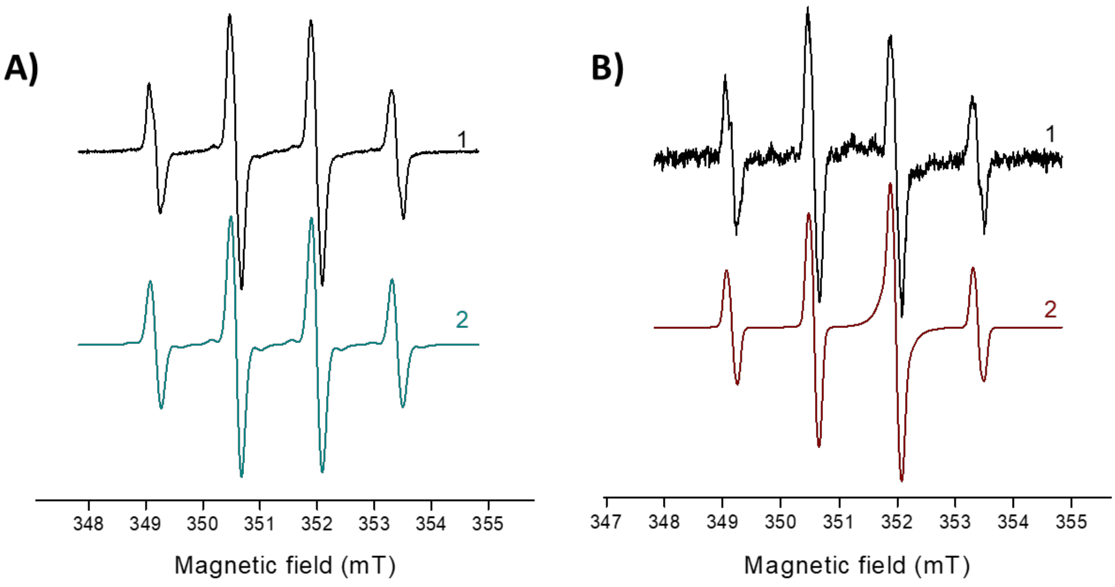

Figure S25*: The normalized experimental (1) and simulated (2) EPR spectra obtained upon continuous in situ LED@400 nm exposure of porphyrin derivatives in chloroform under argon in the presence of DMPO spin trapping agent and NAC:* ***A****)* ***P3*** *and* ***B****)* ***P4****.*

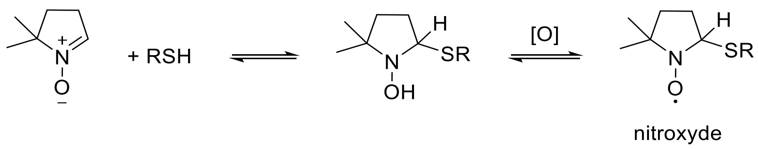

Figure S26*: Forrester-Hepburn mechanism.*

Mechanisms with ROS probe:

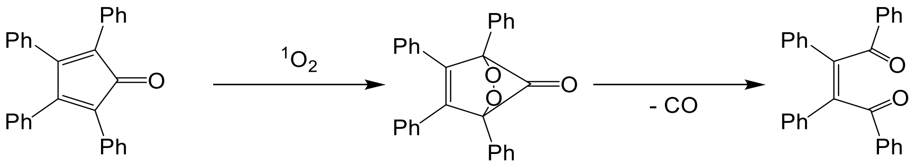

Figure S27*: Oxidation of TPCPD in the presence of singlet oxygen*

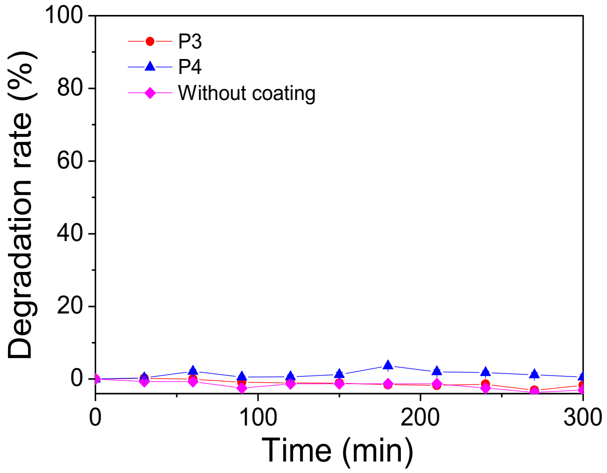

Figure S28*: Adsorption of AR14 on surface of* ***P3*** *(or* ***P4****)-based coating without light. The controls without coating and without light are presented to control the stability of solution.*

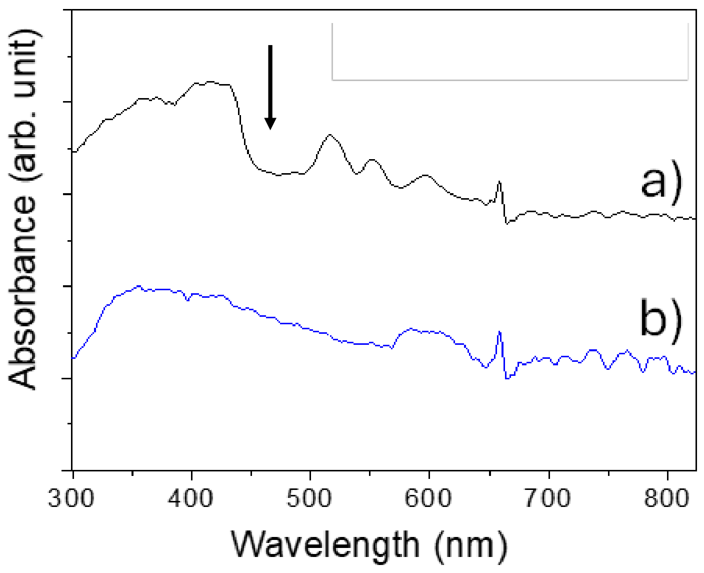

***Figure S29**: UV-Visible spectra of **P4**-based coating a) before and b) after three cycling processes of AR14 photodegradation under UV irradiation (UV lamp, λ_{max} = 365 nm).*

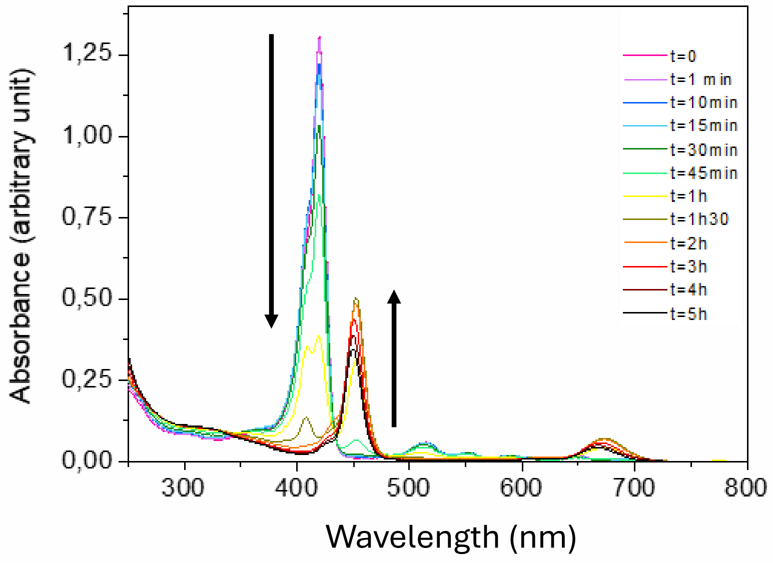

***Figure S30:** Photolysis of a DCM solution of **P4** under LED@405 nm irradiation.*

TABLES

Table S1*: Excitation energies (λ) and oscillator strengths (f) for different excited states transitions of* ***P3*** *and* ***P4*** *determined by TD-DFT using B3LYP/6-31G(d) basis set.*

Porphyrin	transition	λ_{abs} (nm)	*f* (a.u.)	*Contribution*	
**P3**	$S_0 \rightarrow S_1$	580.23	0.0415	HOMO-1 → LUMO	-0.19948
				HOMO-1 → LUMO+1	0.33880
				HOMO → LUMO	0.50985
				HOMO → LUMO+1	0.28713
	$S_0 \rightarrow S_2$	544.40	0.0476	HOMO-1 → LUMO	-0.35824
				HOMO-1 → LUMO+1	-0.19620
				HOMO → LUMO	-0.29011
				HOMO → LUMO+1	0.49743
	$S_0 \rightarrow S_3$	411.48	1.4566	HOMO-3 → LUMO	0.15421
				HOMO-3 → LUMO+1	0.10138
				HOMO-1 → LUMO	-0.26624
				HOMO-1 → LUMO+1	0.49729
	$S_0 \rightarrow S_4$	405.86	1.7718	HOMO-1 → LUMO	0.49817
				HOMO-1 → LUMO+1	0.27610
				HOMO → LUMO	-0.19316
				HOMO → LUMO+1	0.36085
**P4**	$S_0 \rightarrow S_1$	584.96	0.0744	HOMO-1 → LUMO	0.22359
				HOMO-1 → LUMO+1	0.31080
				HOMO → LUMO	0.49065
				HOMO → LUMO+1	-0.33062
	$S_0 \rightarrow S_2$	549.73	0.1146	HOMO-1 → LUMO	-0.32763
				HOMO-1 → LUMO+1	0.21067
				HOMO → LUMO	0.33993
				HOMO → LUMO+1	0.48087
	$S_0 \rightarrow S_3$	413.58	1.7850	HOMO-5 → LUMO	-0.13746
				HOMO-3 → LUMO	0.17887
				HOMO-1 → LUMO	0.10907
				HOMO-1 → LUMO+1	0.54844
	$S_0 \rightarrow S_4$	405.39	1.5069	HOMO-3 → LUMO+1	-0.11532
				HOMO-1 → LUMO	0.55628
				HOMO-1 → LUMO+1	-0.10170
				HOMO → LUMO+1	0.37823

Table S2*: Photochemical properties of porphyrins.* [a] *determined by peak potential using cyclic voltammetry.* [b] *calculated from the wavelength at the intersection of the absorption and emission spectra (λ_{inter}), according to $E_S = 1240/\lambda_{inter}$.* [c] *from the phosphorescence experiments.*

Molecules	E_{ox}[a] (eV)	E_{red}[a] (eV)	E_S[b] (eV)	E_T[c] (eV)
**P3**	1.08	-1,22	1.90	1.43
**P4**	0.87	-0,94	1.92	1.43
CQ	0.12 [1]	-1.25 [1]		2.21 [1]
MDEA	0.72 [2]		-	-
Iod	-	-0.63 [3]	-	-
Cysteamine	0.92 [4]		-	-
N-acetylcysteine (NAC)	0.79		-	-

*Table S3: Optimized ground state coordinates of **P3** obtained at the B3LYP/6-31G(d) level of theory.*

**P3**			
B3LYP/6-31G(d)			
0 1			
C	2.56243300	3.46556500	-0.22017500
C	3.51145500	2.49860800	-0.22001300
C	2.80619400	1.23025100	-0.06760600
N	1.45444300	1.42907300	0.00417100
C	1.28104600	2.78477500	-0.06792000
C	-3.46476100	2.55310500	0.15896800
C	-2.48748600	3.51262000	0.15881100
C	-1.21739600	2.85917200	0.02995800
N	-1.47954100	1.50853200	-0.03423700
C	-2.83428800	1.27118000	0.02990800
C	0.04722500	3.47107400	-0.01628400
C	-2.56240000	-3.46564600	-0.22008300
C	-3.51143000	-2.49870000	-0.21981500
C	-2.80621500	-1.23033300	-0.06743900
N	-1.45444400	-1.42912800	0.00428400
C	-1.28101700	-2.78481500	-0.06784500
C	-3.46748600	0.01709500	-0.01600600
C	3.46482200	-2.55318900	0.15830700
C	2.48754300	-3.51271800	0.15822800
C	1.21744600	-2.85928200	0.02960300
N	1.47956600	-1.50862400	-0.03462300
C	2.83432700	-1.27127100	0.02952400
C	3.46748500	-0.01714900	-0.01625800
C	-0.04722100	-3.47114700	-0.01638200
C	-4.96496500	0.02369800	-0.00529600
C	4.96497200	-0.02371000	-0.00550100
C	0.06810000	4.96909600	-0.00604500
C	-0.06811300	-4.96916500	-0.00606200
C	-5.67717000	-0.51538500	1.07789500
C	-7.07203800	-0.51266400	1.09834800
C	-7.76217300	0.03574500	0.01946400
C	-7.08675400	0.57830200	-1.07142400
C	-5.69154200	0.56951300	-1.07536000
C	5.67705000	0.51524000	1.07785000
C	7.07191500	0.51256700	1.09842900
C	7.76216700	-0.03563000	0.01950600
C	7.08687000	-0.57799400	-1.07154700
C	5.69164900	-0.56926200	-1.07561100
C	-0.46292700	5.70329100	-1.07911300
C	-0.44190700	7.09899700	-1.06815900
C	0.10748800	7.78429900	0.01779300
C	0.63709700	7.06537100	1.09196800
C	0.61851800	5.66986400	1.07962400
C	0.46253400	-5.70345000	-1.07924600
C	0.44151300	-7.09915900	-1.06816600
C	-0.10752900	-7.78437000	0.01801600
C	-0.63679500	-7.06534700	1.09230300

C	-0.61821000	-5.66984600	1.07983700
H	2.70351900	4.53107600	-0.32966600
H	4.57923400	2.62119300	-0.33080700
H	-4.52902700	2.70815800	0.25266000
H	-2.62155600	4.57978100	0.25092600
H	-0.76911900	0.78531500	-0.07800800
H	-2.70350700	-4.53115500	-0.32955100
H	-4.57920900	-2.62129700	-0.33061000
H	4.52911100	-2.70825700	0.25170100
H	2.62165200	-4.57989300	0.25012000
H	0.76912100	-0.78539500	-0.07788200
H	-5.13493100	-0.93559500	1.91959500
H	-7.61068400	-0.92714000	1.94342300
H	-7.63670400	0.99665200	-1.90725600
H	-5.16138400	0.98363800	-1.92783600
H	5.13470400	0.93529400	1.91955700
H	7.61047700	0.92691500	1.94362100
H	7.63691200	-0.99614800	-1.90741600
H	5.16159300	-0.98324200	-1.92822200
H	-0.88387400	5.17445400	-1.92969600
H	-0.85148300	7.64974700	-1.91059400
H	0.12301300	8.87066700	0.02690400
H	1.06247500	7.59010600	1.94313500
H	1.02570300	5.11451000	1.91980500
H	0.88319300	-5.17469900	-1.93002600
H	0.85080800	-7.64997900	-1.91069300
H	-0.12304700	-8.87073700	0.02721700
H	-1.06191700	-7.59001000	1.94364200
H	-1.02513100	-5.11441500	1.92009400
Br	-9.67928300	0.04378900	0.03642100
Br	9.67927500	-0.04360800	0.03663000

Table S4: *Optimized ground state coordinates of* ***P4*** *obtained at the B3LYP/6-31G(d) level of theory.*

**P4**			
B3LYP/6-31G(d)			
0 1			
C	-2.48655400	3.51747400	-0.19002800
C	-3.45751700	2.57234400	-0.19071300
C	-2.78286900	1.28900900	-0.02772100
N	-1.42695700	1.45733400	0.04894800
C	-1.22198900	2.80849600	-0.02811200
C	3.51884700	2.47820900	0.21349700
C	2.56056300	3.45690200	0.21312900
C	1.27839000	2.82937600	0.07662300
N	1.51442500	1.47403600	0.00798800
C	2.86435400	1.20963600	0.07594100
C	0.02661900	3.46727200	0.02713600
C	2.48653700	-3.51749000	-0.19004200
C	3.45751200	-2.57238700	-0.19081600
C	2.78287300	-1.28902200	-0.02788800
N	1.42697100	-1.45732000	0.04882000

C	1.22198100	-2.80847700	-0.02818700
C	3.47370500	-0.05692800	0.02676200
C	-3.51886300	-2.47824800	0.21346200
C	-2.56056200	-3.45692900	0.21306400
C	-1.27840100	-2.82935000	0.07668000
N	-1.51445200	-1.47401800	0.00814600
C	-2.86438100	-1.20965500	0.07607600
C	-3.47370600	0.05691900	0.02691600
C	-0.02662200	-3.46723600	0.02715100
C	4.96940700	-0.08188200	0.03663100
C	-4.96941800	0.08186900	0.03677900
C	0.03770200	4.96544700	0.03786900
C	-0.03770800	-4.96543900	0.03788600
C	5.67588500	-0.67202100	1.09572800
C	7.06881500	-0.69077100	1.10186000
C	7.81421800	-0.12551700	0.05263200
C	7.10205600	0.46387400	-1.00881200
C	5.71183900	0.48651900	-1.01463600
C	-5.67596000	0.67187100	1.09588300
C	-7.06889100	0.69065100	1.10188800
C	-7.81422300	0.12554000	0.05252900
C	-7.10198300	-0.46374500	-1.00891900
C	-5.71176600	-0.48640500	-1.01462000
C	-9.28417100	0.17639200	0.11233000
C	9.28416500	-0.17632300	0.11255000
C	10.15307300	0.30199000	-0.78838000
C	-10.15301300	-0.30216700	-0.78853200
C	0.59020000	5.68882100	-1.03175900
C	0.59915100	7.08465900	-1.02023200
C	0.05863000	7.78123300	0.06300600
C	-0.49228900	7.07329700	1.13377900
C	-0.50359400	5.67771600	1.12070500
C	-0.58984500	-5.68879200	-1.03193100
C	-0.59878600	-7.08463500	-1.02040200
C	-0.05861600	-7.78119000	0.06302400
C	0.49194700	-7.07325400	1.13397700
C	0.50325000	-5.67766600	1.12090000
H	-2.60264900	4.58536300	-0.30589700
H	-4.52160100	2.71779300	-0.30816000
H	4.58557200	2.61109600	0.31238600
H	2.71528000	4.52082000	0.31047300
H	0.79125400	0.76375800	-0.03817600
H	2.60258300	-4.58539900	-0.30577500
H	4.52159800	-2.71780100	-0.30831700
H	-4.58559100	-2.61115000	0.31227100
H	-2.71523600	-4.52086900	0.31021400
H	-0.79129200	-0.76371400	-0.03789300
H	5.12776200	-1.11051700	1.92460600
H	7.59192700	-1.14854400	1.93813800
H	7.63790900	0.90612400	-1.84347900
H	5.18749400	0.93980600	-1.85115700
H	-5.12790900	1.11026300	1.92487200
H	-7.59205200	1.14836800	1.93816500

H	-7.63775000	-0.90587900	-1.84370500
H	-5.18736100	-0.93960700	-1.85114900
H	-9.68271500	0.66482000	1.00149800
H	9.68265000	-0.66451500	1.00187500
H	9.84310000	0.80203600	-1.70222500
H	11.22343400	0.20450500	-0.63407600
H	-9.84297000	-0.80248800	-1.70220200
H	-11.22338600	-0.20462700	-0.63434300
H	1.00449100	5.15142600	-1.88024400
H	1.02512000	7.62681000	-1.86014400
H	0.06640900	8.86769900	0.07264300
H	-0.91105800	7.60666000	1.98288600
H	-0.92737800	5.13082700	1.95822200
H	-1.00389000	-5.15138600	-1.88053000
H	-1.02446800	-7.62680500	-1.86044600
H	-0.06638500	-8.86765700	0.07265900
H	0.91044600	-7.60661600	1.98321900
H	0.92679400	-5.13076200	1.95853000

Table S5: *Rates of polymerization of SOA determined by IR after the first seconds of irradiation under LEDs@385 nm, 405 nm, 455 nm and 530 nm with different photoinitiating systems under laminate (a) and under air (b).*

	**Rates of polymerization (Rp/[Mo])**							
**Photoinitiating systems**	385 nm		**405 nm**		455 nm		530 nm	
**P3**/MDEA	0.34[a]	0.21[b]	**0.90[a]**	**0.64[b]**	0.16[a]	0.08[b]	0.10[a]	0.04[b]
**P4**/MDEA	0.49[a]	0.15[b]	**0.87[a]**	**1.09[b]**	0.21[a]	0.35[b]	0.13[a]	0.06[b]
CQ/MDEA	0.47[a]	np[b]	**0.41[a]**	**0.28[b]**	0.83[a]	0.53[b]	0.11[a]	np[b]
**P3**/Iod	1.38[a]	0.70[b]	**2.01[a]**	**0.99[b]**	0.87[a]	0.17[b]	0.44[a]	0.13[b]
**P4**/Iod	0.95[a]	0.51[b]	**1.59[a]**	**1.13[b]**	0.43[a]	0.09[b]	0.24[a]	0.01[b]
CQ/Iod	0.55[a]	0.14[b]	**1.98[a]**	**1.09[b]**	2.34[a]	1.84[b]	1.61[a]	np[b]
**P3**/cysteamine	0.22[a]	0.16[b]	**0.51[a]**	**0.34[b]**	0.13[a]	0.14[b]	0.03[a]	0.08[b]
**P4**/cysteamine	0.12[a]	0.12[b]	**0.27[a]**	**0.23[b]**	0.13[a]	0.06[b]	0.07[a]	np[b]
CQ/cysteamine	Polymerization at Rt							
**P3**/NAC	0.92[a]	0.28[b]	**1.14[a]**	**1.22[b]**	0.22[a]	np[b]	1.2[a]	np[b]
**P4**/NAC	1.19[a]	np[b]	**1.69[a]**	**1.45[b]**	0.92[a]	np[b]	0.58[a]	np[b]
CQ/NAC	0.31[a]	0.07[b]	**0.75[a]**	**0.23[b]**	0.54[a]	0.32[b]	0.13[a]	np[b]

[a] under laminate, [b] under air, np: no polymerization

EQUATION

Rehm-Weller equation.

$$\Delta G_{\mathrm{s\ or\ T}} = F(E_{ox} - E_{red}) - E_{s\,(or\,T)}$$

Equation S1*: Determination of energy change at singlet ΔG_S or triplet state ΔG_T of the electron transfer between a donor and an acceptor under irradiation. Where F is the Faraday constant, E_{ox} is the oxidation potential of the donor, E_{red} is the reduction potential of the acceptor, $E_{S\,(or\,T)}$ is the transition energy from the excited singlet (or triplet) state of the photoinitiator (**P3** or **P4**) to ground state.*

REFERENCES

[1] H. Marcille, J.-P. Malval, M. Presset, N. Bogliotti, A. Blacha-Grzechnik, V. Brezova, Y. Yagci, D.-L. Versace., *Polym. Chem.*, 2020, **11**, 4237

[2] D. Kim, J. W. A. Stansbury, *J. Polym. Sci. A Polym. Chem.* 2009, **47**, 3131.

[3] F. Schnetz, I. Knysh, D. Jacquemin, S. Abbad Andaloussi, M. Presset, S. Lajnef, F. Peyrot, D.-L. Versace, *Polym. Chem.,* 2024, **15**, 1377

[4] I. El-Hallag, A. O. Al-Youbi, A. Y. Obaid, E. H. El-Mossalamy, S. A. El-Daly and A. M. Asiri, *J. Chil. Chem. Soc.*, 2011, **56**, 837
